# Mind the gut: genomic insights to population divergence and gut microbial composition of two marine keystone species

**DOI:** 10.1186/s40168-018-0467-7

**Published:** 2018-05-02

**Authors:** Katharina Fietz, Christian Olaf Rye Hintze, Mikkel Skovrind, Tue Kjærgaard Nielsen, Morten T. Limborg, Marcus A. Krag, Per J. Palsbøll, Lars Hestbjerg Hansen, Peter Rask Møller, M. Thomas P. Gilbert

**Affiliations:** 1 Natural History Museum of Denmark, Section for Evolutionary Genomics, University of Copenhagen, Øster Voldgade 5-7, 1350 Copenhagen, Denmark; 2 Marine Evolution and Conservation, Faculty of Science and Engineering, University of Groningen, Nijenborgh 7, 9747 AG Groningen, The Netherlands; 3 Department of Environmental Science, Environmental Microbial Genomics Group, Aarhus University, Frederiksborgvej 399, 4000 Roskilde, Denmark; 4 NTNU University Museum, 7491 Trondheim, Norway

**Keywords:** Microbiome, Holobiome, Local adaptive potential, Population genomics, Sand lance, Baltic Sea

## Abstract

**Background:**

Deciphering the mechanisms governing population genetic divergence and local adaptation across heterogeneous environments is a central theme in marine ecology and conservation. While population divergence and ecological adaptive potential are classically viewed at the genetic level, it has recently been argued that their microbiomes may also contribute to population genetic divergence. We explored whether this might be plausible along the well-described environmental gradient of the Baltic Sea in two species of sand lance (*Ammodytes tobianus* and *Hyperoplus lanceolatus*). Specifically, we assessed both their population genetic and gut microbial composition variation and investigated not only which environmental parameters correlate with the observed variation, but whether host genome also correlates with microbiome variation.

**Results:**

We found a clear genetic structure separating the high-salinity North Sea from the low-salinity Baltic Sea sand lances. The observed genetic divergence was not simply a function of isolation by distance, but correlated with environmental parameters, such as salinity, sea surface temperature, and, in the case of *A*. *tobianus*, possibly water microbiota. Furthermore, we detected two distinct genetic groups in Baltic *A*. *tobianus* that might represent sympatric spawning types. Investigation of possible drivers of gut microbiome composition variation revealed that host species identity was significantly correlated with the microbial community composition of the gut. A potential influence of host genetic factors on gut microbiome composition was further confirmed by the results of a constrained analysis of principal coordinates. The host genetic component was among the parameters that best explain observed variation in gut microbiome composition.

**Conclusions:**

Our findings have relevance for the population structure of two commercial species but also provide insights into potentially relevant genomic and microbial factors with regards to sand lance adaptation across the North Sea–Baltic Sea environmental gradient. Furthermore, our findings support the hypothesis that host genetics may play a role in regulating the gut microbiome at both the interspecific and intraspecific levels. As sequencing costs continue to drop, we anticipate that future studies that include full genome and microbiome sequencing will be able to explore the full relationship and its potential adaptive implications for these species.

**Electronic supplementary material:**

The online version of this article (10.1186/s40168-018-0467-7) contains supplementary material, which is available to authorized users.

## Background

A major current focus within marine ecology and conservation is to improve our understanding of the mechanisms governing population genetic divergence and local adaptation across heterogeneous environments. Although gene flow may hamper local adaptation, genetic outlier loci across environmental gradients in several marine fishes imply possible local adaptation despite low overall levels of population genetic divergence [1–3]. In a time during which the planet’s oceans are expected to undergo considerable changes in oxygen, temperature, and salinity levels [4], leading to extensive changes in the conditions of available habitats, an organism’s ability to adapt swiftly to these changes will be vital for its survival. It is therefore particularly important to understand which processes drive the genetic divergence that may be at the basis of ecological adaptation, yet our understanding of these mechanisms is rudimentary. Studying the genetic basis of ecological adaptation in natural populations is particularly difficult when population sizes are limited, because in such cases, random effects dominate over deterministic effects and will prevent the possibility of selection to act. In the marine realm, however, there are many species whose populations have high abundances and span different ecological conditions so that natural selection is expected to dominate over random effects [5, 6]. Accordingly, a number of studies have begun to study adaptive genetic variation across environmental gradients. However, while this growing body of research correlates outlier loci signatures with key environmental parameters, such as salinity and water temperature [2, 3, 6, 7], very few studies have gone beyond standard environmental parameters.

Although the debate about adaptation to different environments is classically viewed at the genetic level, it has recently been argued that an organism’s associated microbiome might also play a role [8]. This argument is nested within the holobiome concept, which views an organism as an entity encompassing not only its own but also its microbial symbionts’ genetic information [9, 10]. Specifically regarding adaptation, Alberdi et al. argued that, given (i) gut microbiome communities can have significant and rapid phenotypic effects on their hosts and (ii) the relatively short time frame of many environmental changes, microbiome community changes may provide an important mechanism for adaptation. While challenging to study directly, the first steps in this direction can come from assessing the variation in host species’ microbial communities against the host species genomic divergence and environmental gradients. Although some studies are considering host genomic-microbial relationships, and even environmental-microbial relationships, few studies so far have attempted to take a full hologenomic approach (both genomic and microbial) across environmental variation.

We explored the potential of a host genomic-microbial approach by assessing both the population genetic and gut microbial variation in two sand lance species along the well-described environmental gradient in the Baltic Sea. The Baltic Sea is a semi-enclosed brackish water basin in Northern Europe, which changes from a nearly limnetic to an almost fully marine environment. Fish species, such as herring [1, 3, 11], cod [2, 12], and three-spined stickleback [13], which have low levels of genomic divergence at neutral genetic markers, have been shown to exhibit substantial levels of divergence at some loci (single nucleotide polymorphisms (SNPs)), which accordingly were inferred to be linked with genomic regions under selection. The divergence at outlying SNPs is likely the result of adaptations in marine species to the brackish conditions in the Baltic Sea after its formation as a marine habitat ca. 8000 years before present [14–16], although other causes, such as a secondary contact zone, cannot be ruled out [17].

Five species of sand lances (fishes of the family Ammodytidae) occur at high abundances in the Northeast Atlantic and adjacent waters. Sand lances are known to be closely associated with specific soft substrates [18] and characterized by high levels of residency and short dispersal ranges [19–21], which likely restrict long-distance gene flow in the absence of physical barriers. Sand lances are keystone organisms as a prey for a large number of marine birds, mammals, and other fish species [22–24]. Sand lances are also targeted by commercial fisheries and thus represent a considerable economic resource [25]. Although these characteristics render them both relevant to the study in the context of marine management and as an interesting potential model organism for studying local adaptation, previous research has principally focused on defining sand lance species and populations using few genetic markers [26–30].

We generated genome-wide SNP data and gut microbiome taxonomic composition data for two ecologically and economically important sand lance species, *Ammodytes tobianus* and *Hyperoplus lanceolatus*, along the North Sea–Baltic Sea environmental gradient. We used the data to firstly estimate the levels of population differentiation across this gradient. Secondly, we tested if the observed population genetic structure correlated with environmental factors, including salinity and sea surface temperature (SST), as well as the relative bacterial composition in the water at sampling sites. Thirdly, we characterized each species’ gut microbiome, its inter-specific variation, and its relationship with both environmental parameters as well as host genomic divergence. Lastly, in light of our findings, we discuss the future potential of how a hologenomic approach may add significantly to our understanding of marine species’ ecological adaptive potential.

## Methods

### Sample collection

**Fig. 1:**
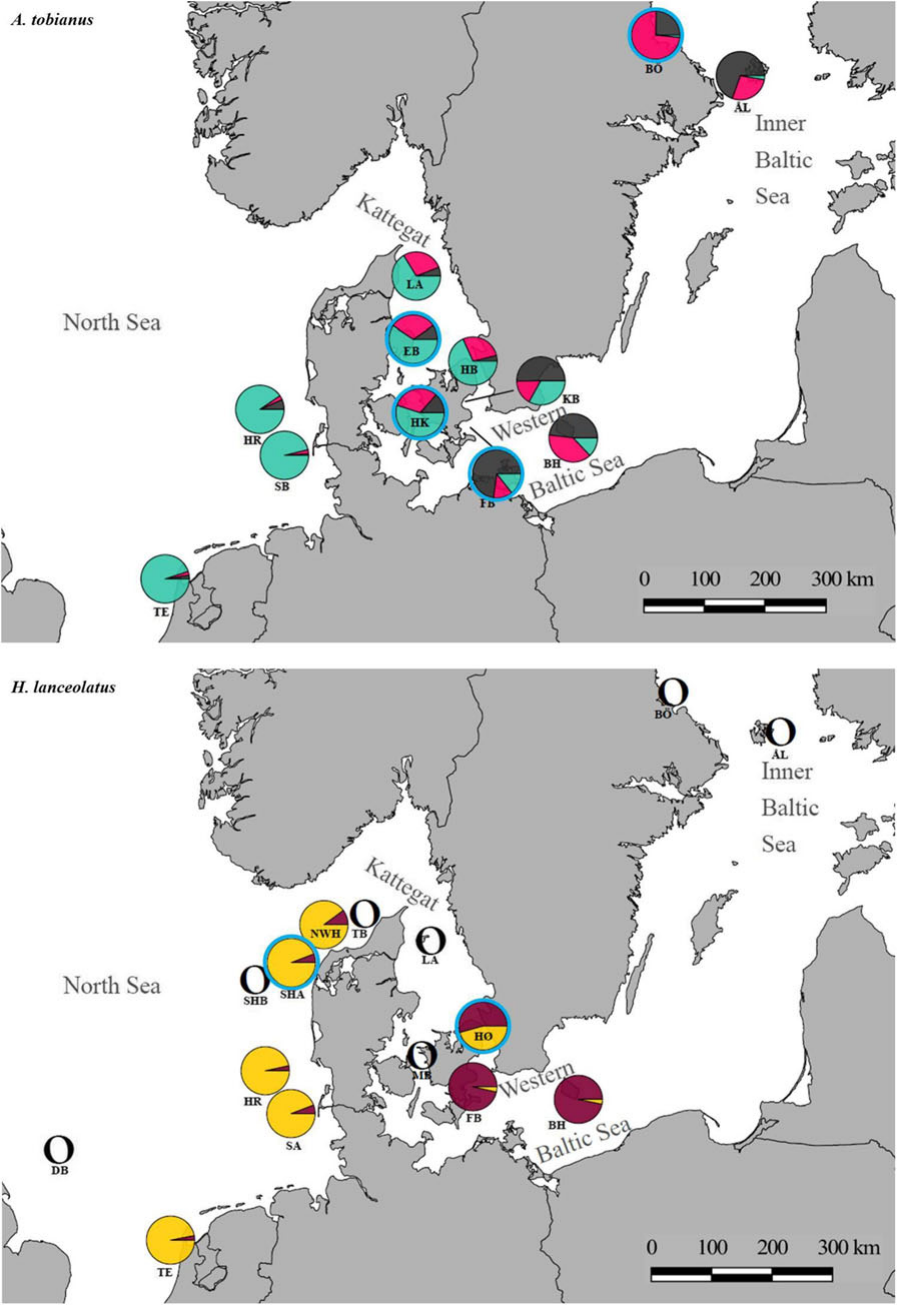
Sampling sites for *A*. *tobianus* (above) and *H*. *lanceolatus* (below). Pie charts represent genetic ancestry proportions per sampling site as estimated in Admixture v.1.3.0 for *K* = 3 (*A*. *tobianus*) and *K* = 2 (*H*. *lanceolatus*). Pie charts encircled in blue indicate sites from which we included 16S data in addition to GBS data. Sampling sites for *H*. *lanceolatus* for which we had < 8 individuals are indicated as hollow circles. TE Texel, SA W-Sylt A, SB W-Sylt B, SHB SW-Hanstholm B, DB Doggerbanke, TB Tannisbugt, HR Horns Rev, SHA SW-Hanstholm A, NWH NW-Hanstholm, LA Læsø, EB Ebeltoft, HB Hornbæk, HØ Helsingør, HK Halsskov, MB Musholm Bugt, KB Køge Bugt, FB Faxe Bugt, BH Bornholm, ÅL Åland, BӦ Bönan

Sand lances were collected at multiple sites across the environmental gradient from the brackish Inner Baltic Sea to the marine North Sea. Samples from *A*. *tobianus* and *H*. *lanceolatus* were collected during May through September 2015 from 12 and 15 different sites, respectively (Fig. 1, Table 1). Individual fishes were collected during commercial and research trawls or caught with near-shore seine nets. Individual muscle tissue samples were collected upon capture (seine nets) or 1–6 h (commercial trawls) post-mortem. If no direct sampling was feasible, individuals were stored in 96% ethanol upon capture and stored at − 20 C° until sub-sampling. Guts were collected under sterile conditions upon capture from a subset of individuals. The samples collected for the gut microbiome analysis were collected from the frontal gut located directly behind the stomach and ending in a tight loop in the gut (hereafter referred to as gut). The external part of the gut was cleaned with sterile equipment to remove any tissue, and the gut wall including contents was used as a sample. Gut samples were stored in 96% ethanol at − 20 C° until further processing.

**Table 1:** Overview of tissue samples for GBS analyses, gut samples for 16S amplicon analyses, and environmental parameters; proportions of major bacterial taxa in the water column are stated as percentage (%) of total composition per sample

Fish species	Sampling site	Sampling date	GBS samples (*n*)	Gut samples (*n*)	Mean # of OTUs	Min # of OTUs	Max # of OTUs	Annual av. sal (PSU)	Annual av. SST (°C)	Annual min SST (°C)	Annual max SST (°C)	SST_Coeff_var (C°)	AP	VM	BC	BP	AB	GP
AT	TE	10.07.15	8					33.47	9.58	− 1.5	21.7	23.2						
	SB	31.07.15	19					31.40	9.90	− 1.1	21.7	22.8						
	HR	05.–06.06.15	15					33.58	12.31	0.3	21.6	21.3						
	LA	29.08.15	27					23.77	9.50	− 1.2	21.9	23.1	27.0	1.6	24.9	2.9	3.3	14.8
	EB	26.09.15	24	5	14.4	10	22	20.73	11.26	− 0.7	23.4	24.1	29.3	2.7	24.4	3.1	3.9	16.5
	HB	03.07.15	29					19.44	11.17	− 0.8	22.6	23.4	29.3	2.7	24.4	3.1	3.9	16.5
	HK	12.08.15	27	10	16.6	9	26	16.54	10.60	− 0.5	23.5	23.9						
	KB	14.07.15	26					9.89	9.90	− 0.2	24.6	24.9	9.9	20.3	13.3	2.9	15.6	2.6
	FB	16.07.15	30	7	21.3	8	35	8.67	9.50	− 0.3	24.8	25.1	9.9	20.3	13.3	2.9	15.6	2.6
	BH	16.07.15	18					7.27	8.48	0.3	22.1	21.8	9.9	27.7	9.5	11.9	10.8	5.0
	ÅL	09.09.15	33					5.83	10.41	− 0.3	23.6	23.9	10.6	16.4	16.6	6.0	18.7	2.9
	BӦ	18.09.15	30	9	40	8	55	5.35	8.18	− 0.2	23.4	23.6	10.1	15.1	17.7	10.0	18.7	4.3
HL	TE	10.07.15	8					33.47	9.58	− 1.5	21.7	23.2						
	SA	03.07.15	17					31.40	9.90	− 1.1	21.7	22.8						
	HR	05.–06.06.15	28					33.58	12.31	0.3	21.6	21.3						
	SHB	23.08.15	1															
	SHA	25.–27.06.15.	14	10	17.1	7	34	33.90	14.23	1	20.4	19.4						
	NWH	30.04.15	17					33.18	10.08	− 0.4	23.2	23.6						
	DB	08.07.15	2															
	TB	24.08.15	1															
	LA	29.08.15	1															
	MB	01.07.15	2															
	HØ	09.09.15	29	9	11.7	5	31	14.29	10.51	− 0.5	22.3	22.7						
	FB	16.07.15	25					8.67	9.50	− 0.3	24.8	25.1						
	BH	16.07.15	25					7.27	8.48	0.3	22.1	21.8						
	ÅL	09.09.15	1															
	BӦ	18.09.15	1															

For sampling site abbreviations, see Fig. 1

*AT A*. *tobianus*, *HL H*. *lanceolatus*, *OTU* operational taxonomic unit, *AB* Actinobacteria, *AP* Alphaproteobacteria, *BC* Bacteroidetes, *BP* Betaproteobacteria, *GP* Gammaproteobacteria, *VM* Verrucomicrobia

### Environmental data

We collected data on salinity, SST, and sea water bacterial taxonomic composition to assess the correlation between genetic data and environmental variables (Additional file 1: Table S1). Salinity and SST data were retrieved from www.smhi.se and www.ices.dk as the annual average salinity during the period 2010 to 2014, as well as the annual, minimum, and maximum average SST during the same period. Data of the relative abundance of six major bacterial taxa in the water column near our sampling sites were obtained from Hu et al. [31]. Although these data were collected in 2013, the long residence time of water in the Baltic basin (3–30 years [32]) and identical sampling season imply that these data likely are broadly representative of the water at our sampling sites.

### Genotyping-by-sequencing (GBS)

#### DNA extraction and sequencing library preparation

Whole-cell genomic DNA was extracted using the KingFisher™ Duo Prime Purification System (Thermo Fisher Scientific Inc., Waltham, USA) following the manufacturer’s protocol for the KingFisher™ Cell and Tissue DNA purification kit (Thermo Fisher Scientific Inc., Waltham, USA). The DNA concentration was estimated using a Qubit™ 2.0 fluorometer (Thermo Fisher Scientific Inc., Waltham, USA). The fragment size range of DNA extractions was estimated for a subset of DNA extractions using an Agilent 4200 TapeStation™ (Agilent Inc.). DNA extractions were subsequently cleaned using the ZR-96 Genomic DNA Clean & Concentrator™ (Zymo Research Inc., Orange, CA, USA) following the manufacturer’s protocol. Population genomic data were generated from the DNA extracts following the GBS approach originally developed by Elshire et al. [33] at the Institute of Biotechnology commercial service (Cornell University, NY, USA) following their standard pipeline [33]. The genomic DNA extracts were digested with the DNA restriction endonuclease *Eco*T221, which has a six-base pair (bp) recognition sequence, and fragments in the size range from 200 to 380 bps were used as the basis for the GBS libraries.

#### GBS sequencing and SNP calling

GBS libraries were sequenced at the Institute of Biotechnology commercial service as single-end 64 bp using an Illumina HiSeq2000™ (Illumina Inc., San Diego, US). Subsequent analytical steps were conducted separately for each sand lance species. Details of data quality filtering and SNP calling are described in Additional file 1.

#### Population genomic analyses

We employed the AMOVA [34] implemented in GenoDive v.2.0 to estimate overall and pairwise levels of genetic divergence as *F*_ST_. We assumed an infinite alleles model [35] and employed 999 permutations to estimate the probability of homogeneity. In order to further assess population genetic structure, we conducted a principal component analysis (PCA) using the smartPCA program in the Eigensoft package [36]. The datasets were reduced to ten eigenvectors, and the principal components 1 and 2 as well as 1 and 3 were plotted using the Perl script Ploteig (Eigensoft package). In order to infer ancestry among sand lances in various areas, we used the model-based approach implemented in the software Admixture v.1.3.0 [37]. Admixture estimations were performed for values of *K* ranging from 2 to 14. Convergence was assumed when the log-likelihood difference among iterations was < 10^−4^. We employed the fivefold cross-validation approach to select the most probable estimate of *K* [38]. The Admixture analysis was undertaken both with and without removing loci that deviated significantly from the expected Hardy-Weinberg genotype frequencies (HWE) under random mating.

#### Detection of outlier loci

We applied three different Bayesian approaches to detect SNPs deviating from neutral expectations and to assess the degree of correlations with environmental parameters. We employed the *F*_ST_-based approach implemented in BayeScan v.2.1 [39] to identify outlier loci. In order to test for associations between population genetic divergence and environmental parameters, we also employed two approaches implemented in BayeScEnv v.1.1 [40] and BayEnv v.2 [41]. Details of the estimations are listed in Additional file 1. In addition, allele frequencies were plotted for outlier loci in order to assess the spatial cline.

### Microbial 16S profiling

#### DNA extraction and purification

Total-cell DNA was extracted from the gut samples using the MoBio Power Soil kit™ (MoBio Laboratories Inc., Carlsbad, USA) following the manufacturer’s instructions. DNA concentrations were quantified using a Qubit™ 2.0 Fluorometer (Thermo Fisher Scientific Inc., Waltham, USA) and normalized to a final DNA concentration at 10 ng/μL.

#### Library preparation and amplicon sequencing

We employed a two-step PCR amplification approach for microbial 16S library preparation. The V3-V4-regions of the bacterial 16S rRNA gene were amplified by PCR using the primers 341F (5′-CCTAYGGGRBGCASCAG-3) and 806R (5′-GGACTACNNGGGTATCTAAT-3) [42]. A subsequent PCR amplification was performed with the Nextera™ XT index primers (Illumina Inc., San Diego, US) in order to attach Illumina MiSeq™ sequence adapters and barcodes to each DNA extract. Full details of 16S library preparation and sequencing are listed in Additional file 1. In the following, the term microbiome refers to the data obtained from the 16S-based libraries.

#### Data filtration and operational taxonomic unit (OTU) clustering

We performed data filtration and clustering in usearch v.8.1.1861 [43]. Details of data filtration and SNP calling can be found in Additional file 1.

#### Gut microbiome taxonomic composition

We employed the QIIME (Quantitative Insights into Microbial Ecology v.1.8.0 [44]) bioinformatic pipeline to estimate the *α* diversity within the sampling sites as well as the Shannon-Wiener [45] and Chao1 indices [46]. The differences in OTU frequencies among sampling sites were assessed using an ANOVA and corrected for multiple simultaneous comparisons using a step-down resampling algorithm described by Westfall and Young [47]. We report all bacteria with *P* < 0.05 at the lowest possible taxonomic level (genus being the lowest level). We further estimated *β* diversity among sampling sites by quantifying the degree of dissimilarities in microbiome composition among sites by a principal coordinate analysis (PCoA) in which we employed a weighted UniFrac distance matrix to account for OTU abundance and phylogenetic ancestry [48]. We tested for significant differences in microbiome composition among sampling sites at the family level using a non-parametric Kruskal-Wallis *H* test [49] and applied the Benjamini-Hochberg False Discovery Rate (FDR) correction to adjust for multiple simultaneous tests [50].

#### Predictors of gut microbiome composition

In order to determine which factors correlate with changes in gut microbiome composition, we implemented various approaches in the R packages vegan [51] and phyloseq [52]. In the case of *A*. *tobianus*, we also included the relative abundances of major bacterial taxa found in the Baltic water as independent parameters in addition to salinity and SST.

We employed a permutational multivariate analysis of variance (Permanova) in the R package vegan [51] using a distance matrix based on Bray-Curtis dissimilarity to identify each variable that has a significant influence on our dataset variation. Results were then plotted by non-metric multidimensional scaling (NMDS) analysis. A biologically plausible combination of significant environmental parameters based on knowledge of the system was fitted onto our unconstrained NMDS ordination.

We then used a constrained analysis of principal coordinates (CAP) ordination implemented in the R package phyloseq [52] to assess which combination of independent variables explained the largest proportion of the variance in gut microbiome composition. To this end, we included those environmental parameters that proved significant in the Permanova described above. Variables were added to the model in order of explanatory variance. In both the NMDS and CAP ordination, collinearity among variables was accounted for by excluding variables varying in a linear manner with variables already added to the model.

#### Assessing the impact of environment versus host genetics upon the gut microbiome composition

We employed an interspecific dataset including the gut microbiome data collected from *A*. *tobianus* and *H*. *lanceolatus*, as well as from a number of Baltic fish species sampled in Køge Bugt during 2016 (listed in Additional file 1: Figure S5). The inclusion of additional fish species served as a reference to contrast the influence of environmental factors with the influence of host genotype on gut microbiome composition. We tested influence of the host species identity with a Permanova and a subsequent CAP ordination with the R package phyloseq [52].

We used microbial composition data collected along a 2000-km transect in the Baltic Sea during 2013 reported by [31] to estimate the degree of correlation between the sand lance gut microbiome and the microbial composition in the water column. We focused our analysis on the relative abundances per sample of non-normalized reads obtained from six bacterial taxa at the phylum and class level, namely, Alphaproteobacteria, Verrucomicrobia, Bacteroidetes, Betaproteobacteria, Actinobacteria, and Gammaproteobacteria. We used a *χ*^2^ test of homogeneity implemented in R [53] to assess the statistical significance of the observed differences in relative bacterial abundance among sampling sites.

## Results

### Genotyping-by-sequencing (GBS)

For *A*. *tobianus*, no individuals were genotyped at more than 20% of the overall identified loci, while no *H*. *lanceolatus* were genotyped at more than 30% of loci (Additional file 1: Figure S1). We excluded all individuals that had more than 90% missing data in either species to keep filtering criteria consistent and to include the maximum possible number of individuals in analyses. Following data filtration, we were left with a final dataset consisting of 4039 SNPs and an overall genotyping rate of 0.976 for *A*. *tobianus* (*n* = 286) and with 4328 SNPs and an overall genotyping rate of 0.980 for *H*. *lanceolatus* (*n* = 163). All analyses were conducted for both species, unless otherwise indicated.

### Population genomic divergence

The global *F*_ST_ was estimated at 0.012 (95% CI 0.011–0.013) in the case of *A*. *tobianus*, and at 0.017 (95% CI 0.015–0.018) in the case of *H*. *lanceolatus*. Pairwise *F*_ST_ estimates among samples of *A*. *tobianus* ranged from 0.001 to 0.041; the highest estimate was observed between the most brackish and the marine sampling sites. The degree of genetic divergence between the geographically intermediate sampling sites and between the brackish and marine sampling sites, respectively, ranged from 0.011 to 0.030 (Additional file 1: Table S2a). The overall pattern of genetic divergence was similar for *H*. *lanceolatus*, with pairwise *F*_ST_ estimates ranging from 0.002 to 0.039. The highest degree of genetic divergence was observed between the Inner Baltic Sea and the North Sea sampling sites (Additional file 1: Table S2b).

**Fig. 2:**
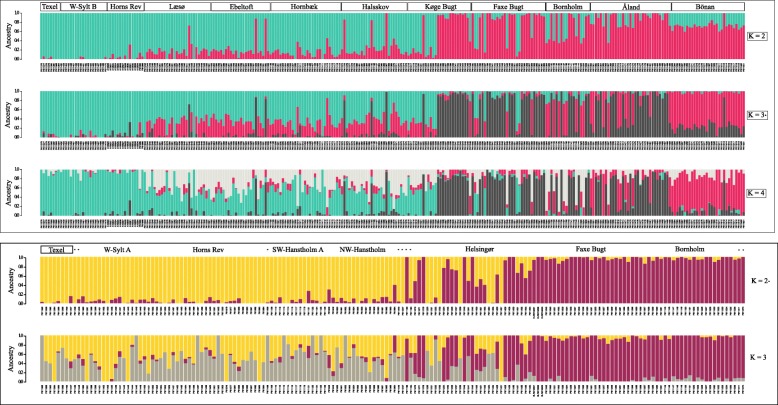
Plots of ancestral fractions from the Admixture cluster analysis for both sand lance species (top = *A. tobianus*; bottom = *H. lanceolatus*). Each vertical bar represents one individual, while the colors indicate the likelihood of this individual belonging to a particular ancestral population. *K* refers to the number of ancestral populations that were assumed to be present in the dataset. *K* = *indicates the most likely *K*. The asterisks in the sampling site heading of *H. lanceolatus* indicates fish from sampling sites with < 8 individuals that were removed for population-level analyses. Samples are sorted from the North Sea (left) to the Baltic Sea (right) sampling sites

The most probable number of genetic clusters was estimated at three and two for *A*. *tobianus* and *H*. *lanceolatus*, respectively (Figs. 1 and 2, Additional file 1: Table S3). The outcome was identical with or without removal of SNPs deviating from HWE (to keep consistent with other studies that used the UNEAK pipeline, we continue with data that included the HWE filtering step). The clustering based upon the PCA yielded the same outcome (Additional file 1: Figure S2).

Overall, we found that the clustering in the case of either sand lance species corresponded well with environmental “regimes.” *Ammodytes tobianus* from marine sampling sites belonged to a different genetic cluster than individuals from Baltic Sea sampling sites (*K* = 3 in Fig. 2). In the Inner Baltic Sea, *A*. *tobianus* consisted of two genetically distinct clusters. *Hyperoplus lanceolatus* shows a similar pattern: individuals in the North Sea sampling sites had pure marine ancestry, while individuals at the two Inner Baltic sites had pure Baltic Sea ancestry (*K* = 2 in Fig. 2). In the following, we will refer to the different clusters as “populations.”

### Loci under putative local adaptation

**Fig. 3:**
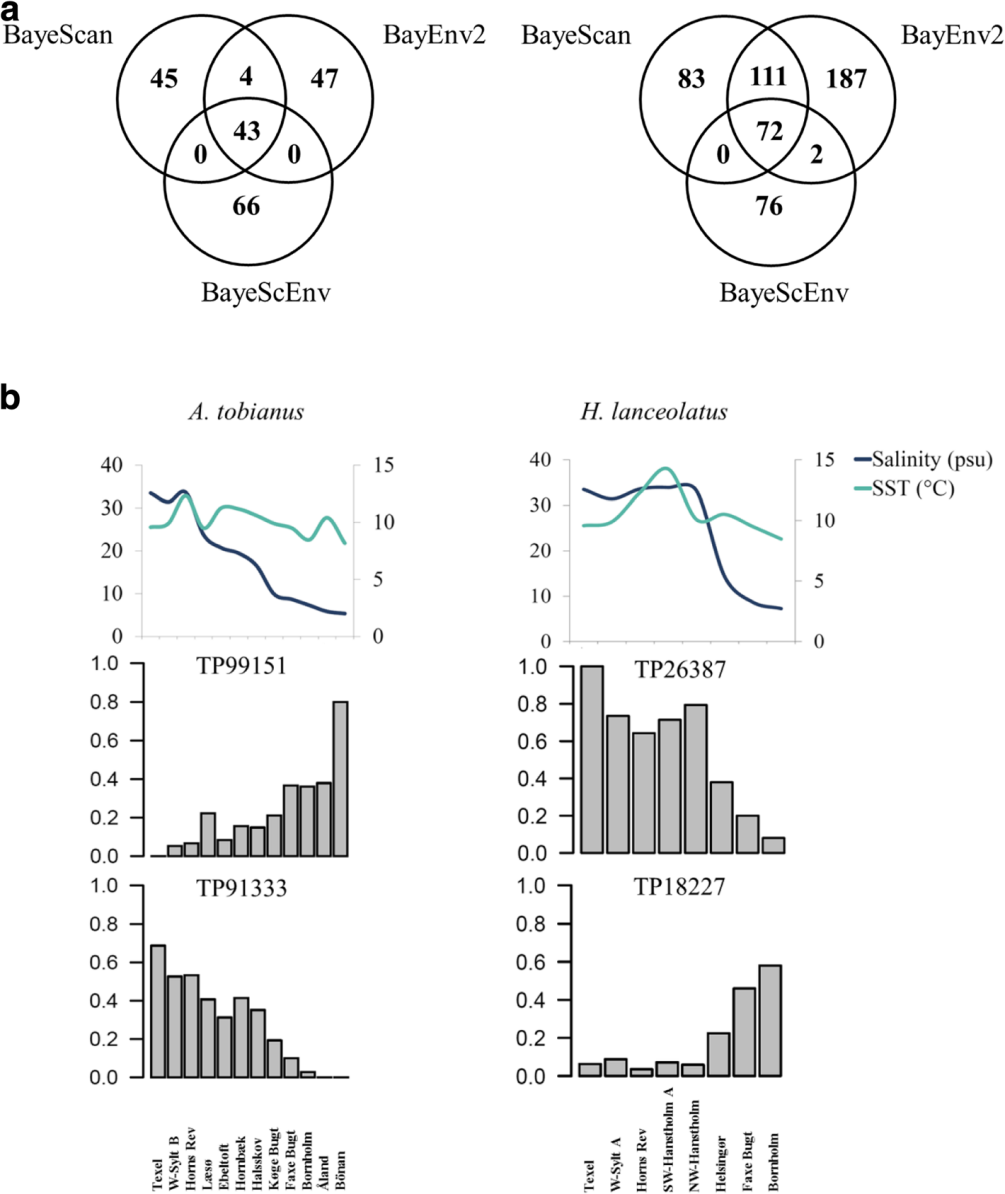
**a** Venn diagram displaying outlier SNPs identified with different outlier detection software (*A*. *tobianus* = left, *H*. *lanceolatus* = right). **b** Allele frequency plots for a subset of four candidate SNPs for divergent selection, as well as sampling-site-specific values for annual average salinity and SST

Our analyses aimed at detecting outlier loci identified numerous SNPs where the spatial change in allele frequencies among sampling sites correlated with the gradient in the environmental parameters included in our analysis. A total of 43 and 72 SNPs were identified as outlier loci in all three of the approaches we applied in *A*. *tobianus* and *H*. *lanceolatus*, respectively (Fig. 3a, Additional file 1: Table S4). Of these, the allele frequencies at 22 and 29 SNPs in *A*. *tobianus* and *H*. *lanceolatus*, respectively, also changed along the environmental gradient, adding further support to the hypothesis that these loci might be subject to selection by factors co-varying with the environment (Fig. 3b, Additional file 1: Figure S3). Although we acknowledge that a clinal pattern such as this could also be expected for a number of neutral loci, we propose that the allele frequency plots presented here represent additional evidence in support of the findings of the outlier analyses. Most of these 22 outlier SNPs in *A*. *tobianus* were correlated with the relative proportions of three major bacterial taxa found in the water (Actinobacteria, Alphaproteobacteria, and Gammaproteobacteria) as well as with the minimum and maximum SST (Additional file 1: Table S4a). The allele frequencies at ten outlier SNPs correlated with the change in salinity, while the allele frequency change in seven SNPs correlated significantly with the change in nearly all environmental parameters. In *H*. *lanceolatus*, 12 of the 29 SNPs were correlated with all tested environmental parameters (salinity and SST) (Additional file 1: Table S4b).

### Microbial 16S profiling

The final microbial dataset consisted of 31 *A*. *tobianus* gut microbiome samples from four sampling sites among which a total of 210 OTUs were detected. We identified 107 different OTUs among 19 *H*. *lanceolatus* gut microbiome samples from two sampling sites (Table 1). The read depths per sample ranged between 1051 and 25,352 in *A. tobianus* and between 1348 and 12,492 in *H*. *lanceolatus* (Additional file 1: Table S5). Both extraction and PCR negative control samples resulted in very low read coverage (≤ 315) suggesting that contamination was negligible and were hence excluded from the final dataset.

### Sand lance gut microbiome composition

**Fig. 4:**
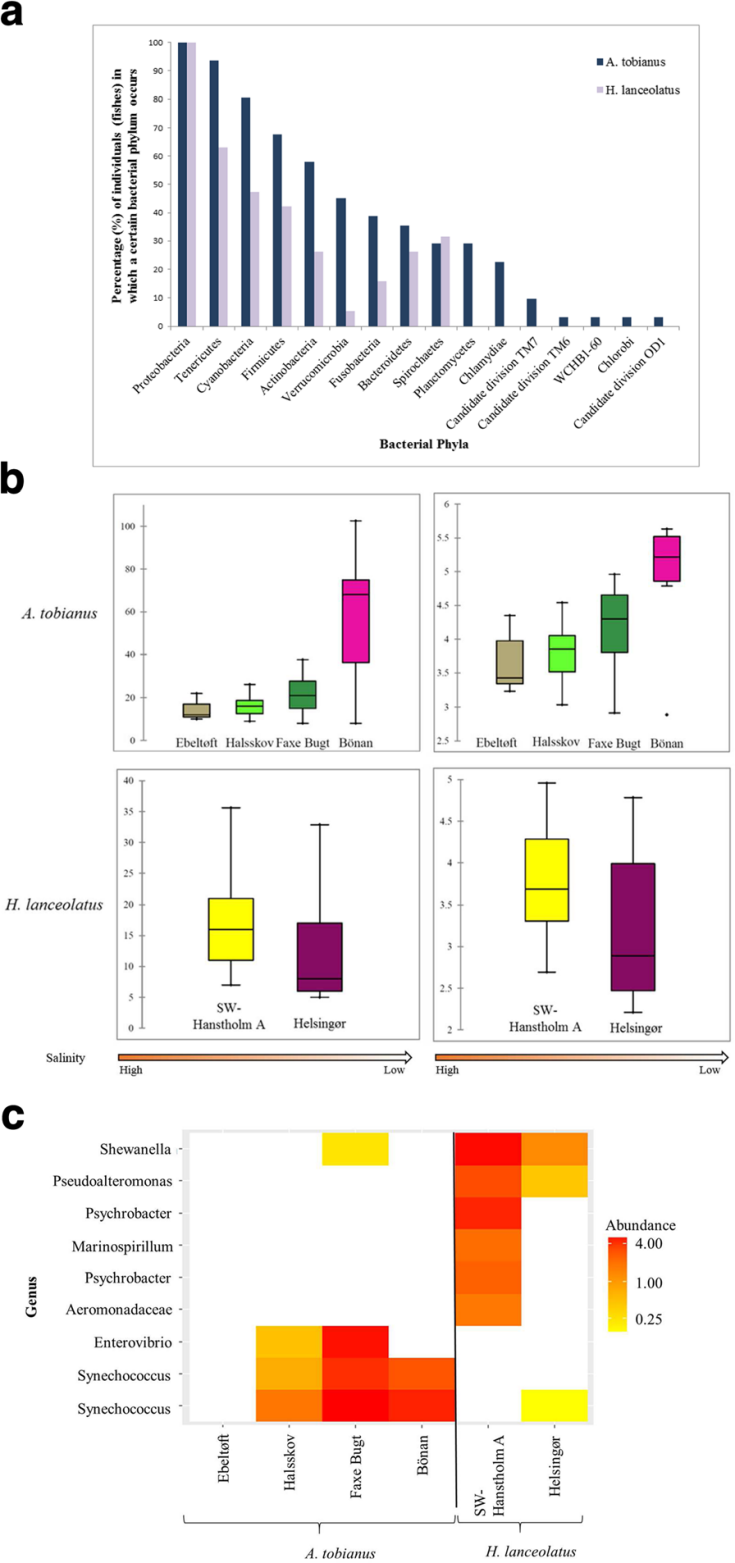
Composition, diversity, and differentiation of *A*. *tobianus* and *H*. *lanceolatus* gut microbial communities. **a** Bar plot depicting the percentage of individuals with the occurrence of the bacterial phyla found in *A*. *tobianus* and *H*. *lanceolatus* guts. **b** Alpha diversity (Chao1 Index left, Shannon-Wiener Index right) increased significantly with decreasing salinity level (indicated as an arrow) in *A*. *tobianus*, while no clear trend is observed in *H*. *lanceolatus*. **c** Heat map of OTUs that changed significantly in relative abundance (%) as a function of sampling site at genus level (*A*. *tobianus* and *H*. *lanceolatus* combined)

At phylum level, the gut microbiome composition of *A*. *tobianus* and *H*. *lanceolatus* was similar and varied only slightly between *H*. *lanceolatus* and *A*. *tobianus* (Fig. 4a). Proteobacteria was the only phylum present in all individuals of both species. The phyla Tenericutes, Cyanobacteria, Firmicutes, Actinobacteria, Bacteroidetes, and Spirochaetes were present in a large percentage of individuals in both species. The largest difference was observed in Verrucomicrobia, which was only present in 5% of *H*. *lanceolatus* individuals but in 45% of the *A*. *tobianus* individuals.

Alpha gut-microbiome diversity was higher overall in *A*. *tobianus* (Chao1 = 8–101; Shannon = 2.89–5.63) compared to *H*. *lanceolatus* (Chao1 = 5–35.6; Shannon = 2.21–4.96). Both the Shannon-Wiener and the Chao1 indices were higher in *A*. *tobianus* gut samples collected at brackish sampling sites compared to marine sites. No such difference between brackish and marine sites was detected between *H*. *lanceolatus* gut samples (Fig. 4b). The relative abundance of four bacterial genera was significantly different among *A*. *tobianus* sampling sites. In comparison, the relative abundance of seven bacterial genera was significantly different between the two *H*. *lanceolatus* sampling sites (*P* < 0.05) (Fig. 4c). Two of these genera belonged to the obligate phototrophic Synechococcus. It is worth noting that only two of these genera (Synechococcus and Shewanella) overlapped between sand lance species.

**Fig. 5:**
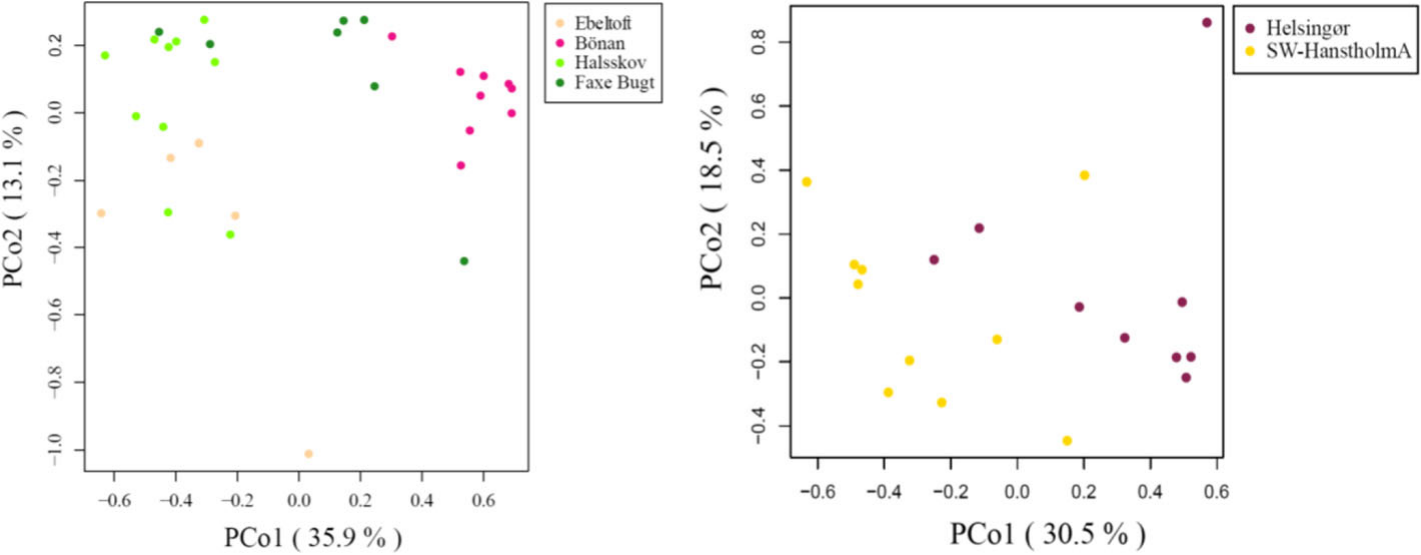
Principal coordinate analysis (PCoA) of the dissimilarity between sand lance gut microbial communities in different sampling sites for *A*. *tobianus* (left) and *H*. *lanceolatus* (right). Color of the circles denotes the sampling site

In the PCoA, the first principal coordinate axis explained 35.9% of variation in gut microbial communities between sampling sites for *A*. *tobianus*, while for *H*. *lanceolatus*, the first axis explained 30.5% of variation (Fig. 5). In *A*. *tobianus*, the level of variation among gut samples from the same sampling site was smaller than that among sites with the exception of Faxe Bugt where three data points group with individuals from other sites. In *H*. *lanceolatus*, the level of variation within and between the two sites was similar.

### Predictors of gut microbiome composition

We tested a range of environmental and host genetic factors to assess how well they explained the observed variance in sand lance gut microbiome composition. The absence of microbial data from the water column at the Halsskov sampling site necessitated the exclusion of this site from the Permanova. We defined the ancestry fraction *Q* of the North Sea group as identified by Admixture for the most likely *K* as the host genetic component for either species. We included this host genetic component as an “environmental parameter” in our analyses. The Permanova identified ten and three environmental variables correlating significantly with the gut microbiome composition in *A*. *tobianus* and *H*. *lanceolatus*, respectively (*P* < 0.001) (Additional file 1: Table S6). The data was visualized in a two-dimensional NMDS ordination to assess possible multivariate interaction of the gut microbiome composition with the environmental parameters (Additional file 1: Figure S4). The main axis among which *A*. *tobianus* sampling sites were separated was characterized by differences in salinity, SST, and the relative abundances of four water bacterial taxa. In *H*. *lanceolatus*, the gut microbiome among sampling sites was not as clearly differentiated, the main axis being characterized by differences in SST, distance, and date, describing the same variation in opposite directions (Additional file 1: Figure S4, Table S6).

**Fig. 6:**
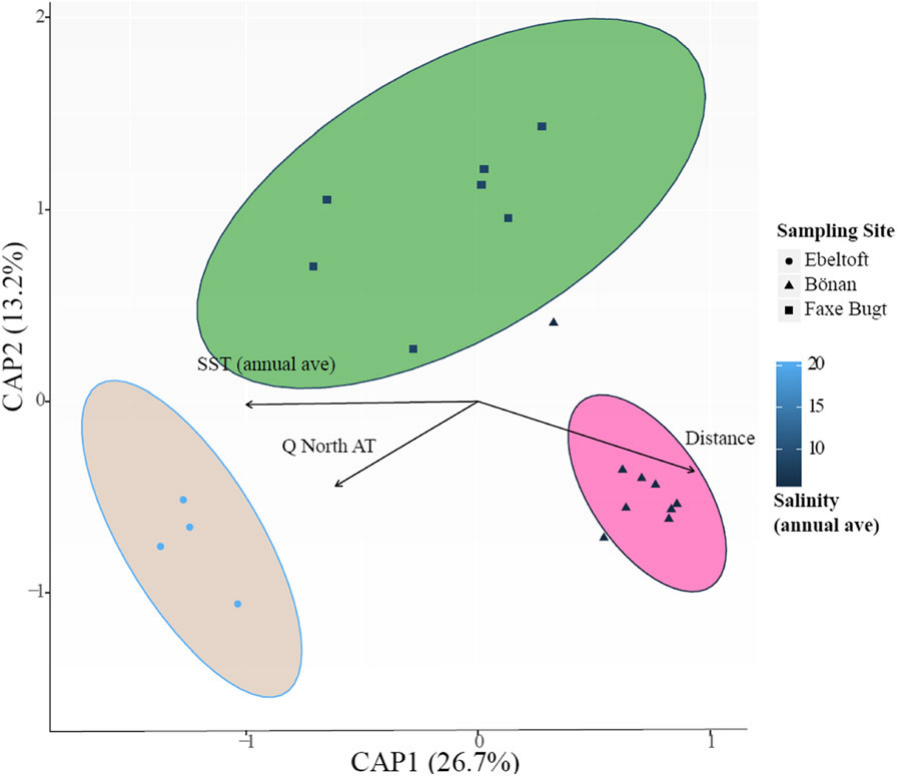
Results of CAP analyses displaying the combination of environmental parameters explaining the largest amount of variation in gut bacterial communities in *A*. *tobianus*. The shape of symbols indicates the sampling site while the color of symbols indicates the salinity

We used CAP ordination to test for statistical significance in the multivariate interactions among environmental parameters and gut microbiome composition. In *A*. *tobianus*, the combination of variables accounting for a significant proportion of the variance in the gut microbiome composition included SST, geographic distance from the westernmost sampling site, and the host genetic component *Q* (CAP1 = 26.7%) (Fig. 6). The one-dimensional ordination in *H. lanceolatus* explained 19.8% of the variance (results not shown).

### Impact of environment versus host genetics on the gut microbiome composition

We conducted a CAP ordination on a multi-specific dataset in order to extend our understanding of the potential influence of host species factors on gut microbiome composition. This revealed that the host species identity was a key explanatory variable for gut microbiome composition (Permanova *P* < 0.001; PC1 = 6.5%, PC2 = 5.5%) (Additional file 1: Figure S5). We then displayed the gut microbiome composition of multiple species from three adjacent sampling sites to illustrate the different scales of similarity in gut microbiome composition among species and sampling sites (Additional file 1: Figure S6). We therefore chose *A*. *tobianus* samples from two sites 131 km apart and samples from an outgroup of fishes (sand goby (*Pomatoschistus minutus*), flounder (*Platichthys flesus*), stickleback (*Gasterosteus aculeatus*)) from a geographically intermediate sampling site (Køge Bugt). Our hypothesis was that gut microbiome composition would be more similar within than between host species, even if individuals of the same species were sampled at different sites. With the exception of a single individual (ZMUC P611035, from Faxe Bugt), the gut microbiome composition was visibly less variable among *A*. *tobianus* across sites than it was among *A*. *tobianus* and other fish species from sites that are geographically closer to each other.

**Fig. 7:**
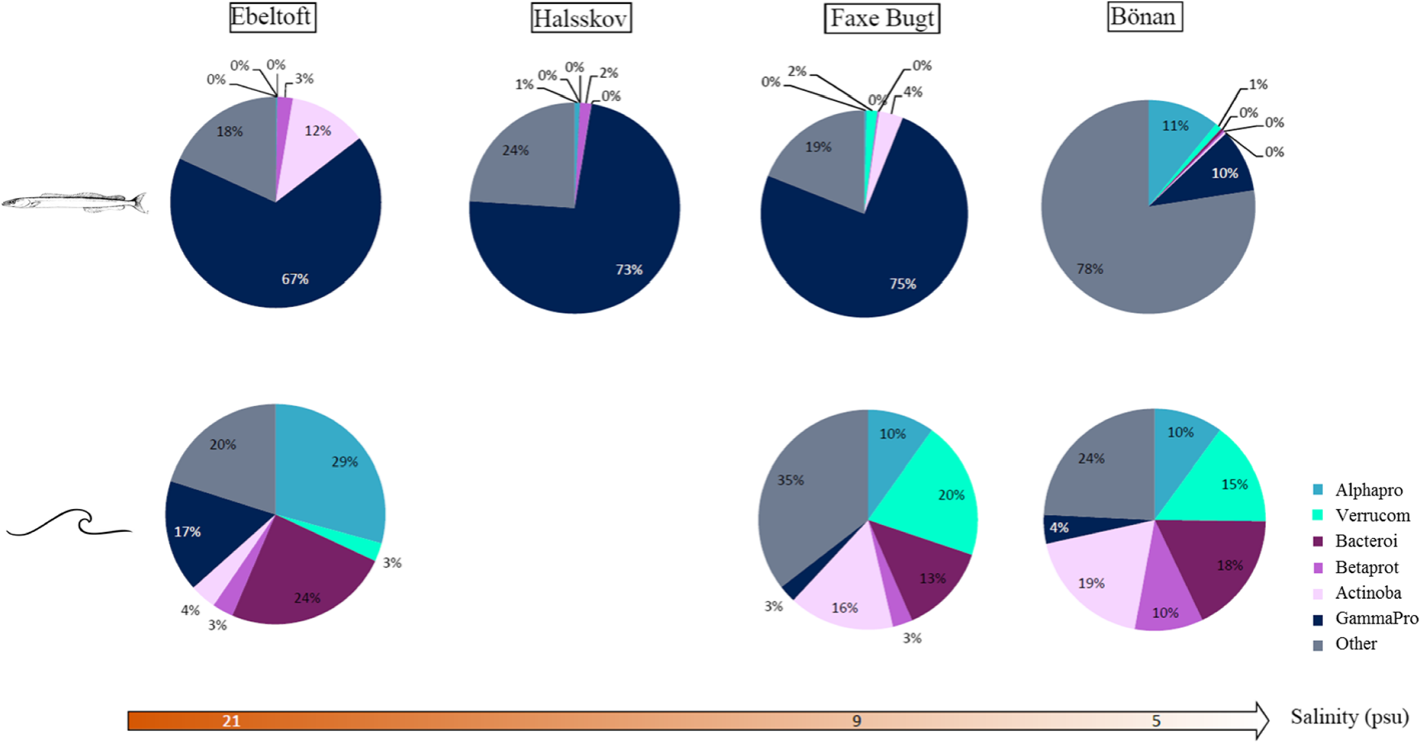
Relative abundance (of non-normalized read numbers) of the six most abundant bacterial taxa at phylum and order level of *A*. *tobianus* guts (above) and in environmental (below) water samples [31]

Correlations between gut microbiome composition and relative abundance trends of bacterial taxa in the environment revealed significant changes in the relative abundances of some of the major bacterial taxa along the North Sea–Baltic Sea environmental gradient (Fig. 7, Additional file 1: Table S7). While some bacterial taxa demonstrated identical abundance trends in gut and environment, others showed opposing relative abundance trends. Specifically, trends were identical in Gammaproteobacteria which became proportionately less dominant in both environmental (water) and gut samples as environmental salinity decreased (Fig. 7). In Alphaproteobacteria, relative abundance increased in guts and decreased in the environment, whereas the opposite trend was observed in Actinobacteria (Fig. 7).

## Discussion

Our findings not only have relevance for the population structure of two commercial species, but also provide insights into potentially relevant genomic and microbial factors with regards to sand lance adaptation across the North Sea–Baltic Sea environmental gradient. Furthermore, our findings provide evidence that sand lance gut microbial communities are influenced by both host genetics and environmental parameters.

### Isolation of Baltic Sea sand lances

The general level of population genetic divergence observed in sand lance was similar to those previously observed in other marine fishes (e.g., [2, 54]). In both sand lance species, the highest degree of genetic divergence was observed between the sampling sites in the North Sea and the Inner Baltic Sea, similar to the population genetic divergence observed in, e.g., Atlantic cod [2], herring [1], flounder [55], and sprat [54]. The proportional change in genetic divergence was highest between sampling sites located in the areas with the steepest change in environmental parameters, such as salinity. Similar qualitative changes in genetic divergence have been reported previously in several other Baltic Sea fish species (e.g., Fig. 2 in [54]). Sand lances in the North Sea tend to be resident and associated with specific habitat types [19]. Consequently, most dispersal is likely to occur during the larval phase, but even this is thought to be limited [20, 21]. Assuming that these dispersal characteristics are shared with Baltic Sea sand lances, they match the genetic divergence between these two seas.

### *Ammodytes tobianus* in the Baltic Sea consists of two genetically differentiated populations

*Ammodytes tobianus* in the Baltic Sea—mainly between Køge Bugt and Åland in the Western to Inner Baltic Sea—seems to belong to two genetically differentiated populations. We suggest that this observation may be attributed to either spatial or temporal segregation. Spatial segregation would occur if different breeding stocks left their spawning areas and mixed at sampling sites outside the spawning season. As we did not sample during the spawning season, we cannot exclude this option. We find spatial segregation unlikely, however, given the sand lances’ residential behavior and limited dispersal capacity [20, 21]. Another possible cause for the observed genetic pattern may be temporal segregation. Temporal segregation would occur if different spawning types were sympatric but reproduced at different times [56]. Populations of *A*. *tobianus* may indeed consist of two distinct but often sympatric spawning types, an autumn and a spring spawning contingent [57] that are known to occur together, e.g., off the coast of West Ireland [58]. These spawning types generally differ from each other in the mean number of vertebrates which is higher in the autumn group [59]. As early as 1934, it was suggested that *A*. *tobianus* in the Baltic Sea consists of two spawning types [60]. More recently, [61] investigated *A*. *tobianus* in the Southern Baltic Sea in the Gulf of Gdansk and based on vertebral counts assigned the individuals of this area to the autumn-spawning component of the stock. While we did not find significant differences in vertebral counts in our fishes that would support a hypothesis of different spawning types (data not shown), we hypothesize that the two genetic *A*. *tobianus* Baltic populations that we detected here may nonetheless represent two sympatric spawning types. In order to gain certainty about co-occurrence of different spawning types in the Baltic Sea, future studies will have to sample individuals during the respective spawning season (autumn and spring) and investigate the presence of ripe gonads in adult individuals. The presence of different spawning types in the same habitat is also known from other Baltic species such as herring [11, 56]. Our results highlight the power of using genetic markers as a tool to monitor the relative proportion of the two spawning types in fishery landings.

### Sand lances along the North Sea–Baltic Sea environmental gradient display signatures consistent with local adaptation

As a geologically young sea that has undergone extreme changes in environmental conditions in the last 8000 years, the Baltic Sea has served as a popular model to assess divergence along an environmental gradient in marine organisms [62]. Our study supports previous work hypothesizing that marine fish populations show signatures of potential local adaptation along the Baltic Sea–North Sea gradient [2, 63, 64]. Given the relatively small number of SNPs, the lack of a reference genome, and the fact that we aimed to exercise great care to avoid over-interpreting our results, we refrained from blasting outlier loci and chose to instead focus on drawing conclusions from the observed correlations and allele frequency plots. In both *A*. *tobianus* and *H*. *lanceolatus*, we found elevated levels of genetic divergence at loci that correlated with SST and salinity (we discuss the relationship with the microbial communities in the water column below). Fishes have evolved physiological mechanisms that differ fundamentally between high- and low-saline environments in order to maintain an internal salinity at 9 PSU [65]. Divergent selection, especially at loci that are associated with salinity tolerance, may therefore be expected to promote reproductive isolation between Baltic Sea and North Sea populations. In a study on Atlantic cod, Berg and colleagues identified outlier SNPs in the cod genome where the population genetic divergence correlated with a salinity gradient as well. The divergent SNPs were located within genes that were known to be associated with osmoregulation and oocyte development. Accordingly, [2] argued that the divergent alleles were the result of selection to a low-saline environment. The results of acclimation experiments and molecular phenotyping in tilapia (*Oreochromis mossambicus*), a euryhaline cichlid, led [66] to infer that osmoregulatory stress could affect gill development. Here, we hypothesize that similar mechanisms related to physiology, egg, and larval survival in low-saline waters likely are the underlying causes of the elevated levels of population genetic divergence between sand lances in the Baltic Sea and North Sea. Ambient temperature is important in affecting metabolic reactions especially of poikilotherm organisms such as fishes. Allele frequencies in a set of genome-wide SNP loci, for example, showed parallel temperature-associated clines in Atlantic cod on either side of the Atlantic Ocean [67]. The observation that the degree of population genetic divergence in multiple of the sand lance outlier loci was significantly associated with salinity and water temperature led us to the hypothesis that environmental heterogeneity in these, or correlated, factors may be an important driving force behind the genetic divergence between the Baltic and North Sea sand lance populations. We acknowledge that spatial replicates will be needed to be able to exclude the possibility that the observed pattern is purely a result of the nature of the data.

### Gut microbiome composition and diversity

We incorporated data of gut microbiome composition from a subset of specimen in each sand lance species to explore associations between host genomics and environment upon the gut microbiome composition. At a basic level, the sand lance gut microbial community composition was comparable to that reported in other fishes [68–70]. Members of the Proteobacteria were the most common phylum observed in the gut microbiome in both sand lance species. Among other common microbial phyla were Actinobacteria, Tenericutes, and Cyanobacteria. Proteobacteria are one of the major phyla in the guts of many other studied fish species (as are Actinobacteria, Tenericutes, and Cyanobacteria) and are found in high abundance in fish guts [68–71]. The microbial taxa observed in the sand lance guts are taxa that aid in nutrient absorption and homeostasis [72]. For example, Proteobacteria help degrade and ferment complex sugars. Actinobacteria assist in maintaining host homeostasis and in inhibition of Gram-negative pathogens and lactic acid fermentation. Tenericutes aid nutrient processing [72].

Microbial diversity estimates in both sand lance species varied substantially among individuals. The microbial diversity was overall higher in *A*. *tobianus* compared to *H*. *lanceolatus*. Although gut microbial diversity is likely correlated with diet [73, 74], we did not include dietary analyses in our study. The relationship between the diet and gut microbial flora is not simple: some authors found that a more diverse diet will increase gut microbial diversity [75], whereas others have reported an inverse relationship in some fishes [74]. The more general observation is that diversity of fish gut microbial communities likely is influenced by multiple environmental variables. Our results suggest that the diversity of the gut microbiome increases with decreasing salinity in *A*. *tobianus*. However, inclusion of diet analysis and additional spatio-temporal replicates in future studies will help us better understand the processes shaping this pattern.

### Microbial communities of the sand lance gut are not mere reflections of environmental microbial communities

Numerous studies across a wide range of organisms, from ants [76] over fishes [77] through humans [78, 79], have shown that the composition of the core gut microbiome is not a simple function of the environment but correlates with the host genetics as well. The gut microbial composition may be a product of host phylogenetic affinities and/or the host’s ecology, both of which are likely to determine the mode of microbiome acquisition, i.e., by vertical transmission and/or from the environment [72].

We assessed the potential differences among fish species and their gut microbial community composition in order to gain insights into possible drivers of the gut microbiome composition. Our analysis indicated non-random differences in gut microbiome composition among different fish species. However, our analysis did not account for variation in microbial communities among sampling sites. Consequently, we cannot attribute the observed differences to key and specific variables, such as spatial heterogeneity, fish age, or prey. In order to account for this caveat, we visualized the gut microbiome composition of *A*. *tobianus* from a subset of sites, in addition to samples from other fish species from a geographically intermediate site. As expected, the intra-specific gut microbial composition was more similar within species compared to that among species (Additional file 1: Figure S6). Our results suggest that a potential influence of host genetic factors on gut microbiome composition depends on the bacterial taxon under investigation. The host genetic component *Q* was among the parameters that best explained observed variation in gut microbiome composition. On the other hand, the presence of obligate photoautotrophic bacteria (Synechococcus) suggests at least partial bacterial uptake from the environment. In our comparison of relative abundance changes of major bacterial taxa between fish guts and surrounding water along the North Sea– Baltic Sea environmental gradient, Gammaproteobacteria showed identical relative abundance trends in guts and water. This implies that the relative abundance of Gammaproteobacteria in sand lance guts may be driven by their relative abundance in the surrounding environment. In Alphaproteobacteria and Actinobacteria on the other hand, gut-bacterial relative abundance trends were converse to those of the surrounding water, suggesting that relative abundances in these taxa might depend more strongly on host species.

Last, we expanded our environmental dataset in the population-genomic outlier analyses of *A*. *tobianus* to include—besides SST and salinity—the proportional abundance of major microbial taxa in the gut microbial samples. The relative abundance of Actinobacteria, Alphaproteobacteria, and Gammaproteobacteria correlated significantly with nearly all detected outlier loci. Actinobacteria are known to be part of the gut, mucosa, and skin in fishes, while Proteobacteria are thought to be the dominant phylum in the guts of many fishes. Among the Proteobacteria, Gammaproteobacteria typically break down and ferment complex sugars and provide particularly important digestive roles [72]. The results of our outlier analysis may indicate that bacteria adopted from the environment play important roles in a population’s adaptability to its local habitat.

However, our study design prevented us from identifying functional aspects, e.g., how salinity and SST correlated with the microbial community in the water column in the Baltic. Controlled laboratory experiments would be needed in future studies to differentiate between correlations of environmental bacteria and an organism’s gut microbiome alone and/or with additional environmental parameters, ultimately fully linking the system together. The combination of in situ work and common garden experiments seems particularly promising for moving beyond the detection of correlations and to assess the causal roles of microbiome and environmental factors in shaping the adaptive potential of wild vertebrate species. However, taken together, our results support the notion that the gut microbial community composition is, in part, a function of the host’s genomic background.

### The potential of combining population genomics with gut microbial data to gain insight to an organism’s ecological adaptive potential

It has recently been suggested that in order to cope with rapidly changing environmental conditions, organisms may rely on high phenomic plasticity conferred by their gut bacterial symbionts [8]. Changes at the genetic level that bring about evolutionary change often need many generations to be established in a population. For vertebrates, with their slow reproductive strategies and long generation times, this can be insufficient for short-term adaptation and survival. Phenotypic plasticity may hence be an essential factor in determining how well vertebrates adapt to fast environmental change [8]. Among host-associated microorganisms, the bacteria of the gut are thought to be the most influential symbiotic community, affecting health, immunity, digestive metabolism, and consequently fitness of their hosts [80–82]. If indeed an organism’s adaptive potential may be enhanced through help of its gut microbiome, it stands much better chances of survival when faced with the need to adapt swiftly. Given this putative central role of the gut microbiome, knowledge of such communities is a vital addition to population genomics in studies of local adaptation [72, 83]. Recent research investigating the genetic basis of microbes (the “who”) hypothesizes that bacteria might confer certain abilities or tolerances to their hosts. However, only very few studies have explicitly tested whether certain bacteria actually do confer such abilities or tolerances (the “how” and “why”) [84]. In fishes, microbiome research has mainly focused on lab-reared [71, 85, 86], captured [74], or aquacultured fish [87], while to date there is only a limited number of studies on wild populations and only on few economically important species such as salmon [69]. From few studies involving wild populations, we know that inferences about the gut microbiome made from captive animals may not always be transferred to their wild counterparts [70, 88]. In this sense, studies of wild non-model organisms are invaluable to gain a better understanding of how the gut microbiome and host act together as an entity in facilitating rapid ecological adaptation. In this study, we for the first time use both population genetic and gut microbial data to make inferences about the population genetic divergence of two fish species in their natural habitat. Our study may serve as a benchmark for future work that aims to integrate population genomic with gut microbial data to investigate an organism’s ecological adaptive potential.

## Conclusions

In this study, we take a new approach by using population genomic and 16S gut microbial data to shed light onto the population genetic divergence of two closely related non-model fish species along an environmental gradient. Three major findings emerge from our study: First, the Baltic Sea harbors unique genetic populations of sand lances that are differentiated from the North Sea. We discovered that genomic regions showed elevated divergence not only as a potential response to salinity- and SST-related natural selection, but that these regions also correlate with the relative bacterial composition of the water. This could hint at a potential influence of environmental microbes on the adaptive genetic divergence of these marine fishes. Second, we confirmed that *A*. *tobianus* in the Baltic Sea exists as two genetic stocks co-occurring in the same habitat. This sparks interest in adapting future fishery management measures. Third, the gut microbial communities of sand lances are not a mere reflection of environmental microbes, but rather the fishes seem to excerpt some degree of internal control and selection.

Recent insights into the extent of microbial influence on an organism’s well-being and fitness suggest that the potential of individuals, populations, and species to genetically adapt to changing environments may not be governed by the interactions of these entities with the environment alone. Rather, bacteria seem to partake in forming the adaptive potential of many organisms by composing part of their holobiome. In this sense, population genomic studies aimed at understanding a species’ local adaptive potential will miss part of the story when not considering bacterial communities.

We are only beginning to understand how the interplay among hosts, their microbes, and the environment regulates ecological adaptation. One can imagine that in the future, as genomic technologies become cheaper and more readily applicable, our study may serve as a benchmark to design yet more rigorous approaches to learn what enables an organism to survive and adapt in a rapidly changing environment. The combination of in situ work and common garden experiments seems particularly promising for moving beyond the detection of correlations and to assess the causal roles of bacterial and environmental factors in shaping the adaptive potential of wild vertebrate species.

## Additional file

Additional file 1: Supplementary Material. (DOCX 13909 kb)
