# Supplementary material for: Mind the gut: genomic insights to population divergence and gut microbial composition of two marine keystone species
Source: Microbiome. 2018 May 2;6:82. doi: 10.1186/s40168-018-0467-7 (PMC5932900; doi:10.1186/s40168-018-0467-7)
Supplement: Supplementary file 1 — Supplementary Material. (DOCX 13909 kb) [file 40168_2018_467_MOESM1_ESM.docx]

**Table S1: Environmental parameters that were included in GBS and 16S studies.**

| **Parameter** | **Measurement** | **Details of Recording** |
| --- | --- | --- |
| Date | Julian Day | - |
| Sampling Site | Distance | For each species, we appointed the most Westerly sampling site as 0, and calculated distances of all other sampling sites respective to this first site. |
| Salinity (PSU) | ICES: www.ices.dk SMHI: http://opendata-download-ocobs.smhi.se/explore/?parameter=4# | Annual average over five years (2010-2014) |
| Sea Surface Temperature (SST) | ICES: www.ices.dk SMHI: http://opendata-download-ocobs.smhi.se/explore/?parameter=4# | Annual average over five years (2010-2014)* Minimum average over five years (2010-2014)* Maximum average over five years (2010-2014)* Coefficient of variance in SST (2010-2014)* |
| Baltic Sea water metagenomic data | Abundance of six most abundant bacterial phyla and classes in the water column, data from Hu et al. 2016 | Methods detailed in Hu et al. 2016** |
| Host genetic variable*** | The ancestry fraction Q of the North Sea population | Individual-specific Admixture output |

* for Bönan only 2013-2014 were used as reference (no quality control of data available before this time) (SMHI pers comm)

** [[1](#_ENREF_1)]

*** included as an independent parameter in 16S amplicon analyses

**GBS data filtration and SNP calling**

Raw GBS reads were de-multiplexed and filtered using the UNEAK3 pipeline [[2](#_ENREF_2)]. Tags were defined as groups of >5 identical reads in the UMergeTaxaTagCountPlugin. In order to minimize false SNP calls while maximizing the number of generated SNPs, we kept the error tolerance rate in the UTagCountToTagPairPlugin at the default 0.03, and the minimum minor allele frequency (MAF) in the UMapInfoToHapMapPlugin at 0.05. In order to decide on a minimal amount of accepted missing data per individual while including the maximum number of individuals in analyses, we investigated SNP coverage in our UNEAK-filtered dataset. Following UNEAK filtering, data were re-genotyped using an in-house script (available from the authors upon request) with the following settings: only sites with a genotype probability ≥90% and a phred score ≥30 were called as SNPs. Further, all SNPs with >2 alleles were removed, as were SNPs that deviated significantly from the expected Hardy-Weinberg genotype frequencies (HWE) under random mating in >75% of sampling sites. Lastly, we removed all SNPs with >10% missing data using Plink [[3](#_ENREF_3)] and for all population-level estimates excluded any sampling sites with < 8 individuals.

**Signatures of local adaptation and association with environmental parameters**

We detected loci deviating from the neutral model with an *F*_ST_-based approach in BayeScan v.2.1 [[4](#_ENREF_4)]. To test model consistency, we used prior odds for the neutral model of 10, 100, 1,000 and 10,000 and compared results in a preliminary step. For the final analyses, we used prior odds for the neutral model of 100 as recommended in the BayeScan v.2.1 manual. In order to tune the acceptance rates of posterior distributions, we used 20 successive pilot runs of 5,000 iterations length each. We conducted three independent analyses per species with a thinning interval of 20 and 100,000 total iterations following a 100,000-iteration burn-in period. We used Geweke’s convergence diagnostic to ensure convergence of each MCMC analysis [[5](#_ENREF_5)], and the Gelman diagnostic to monitor the convergence of our three independent MCMC outputs [[6](#_ENREF_6)] implemented in the R package coda [[7](#_ENREF_7)].

To test for associations between population genetic differentiation and environmental parameters, we employed two additional approaches. Among the outlier detection methods that take environmental variables into consideration, BayeScEnv is particularly well suited for species with high dispersal rates [[8](#_ENREF_8)]. We used the software with a prior preference *p* for the locus-specific model = 0.2 as the ideal trade-off to have high power while keeping the false positive rate acceptable, as has been shown to be most appropriate for species with high dispersal rates [[8](#_ENREF_8)]. The prior preference for the non-neutral model was set to 0.01 in order to be comparable to our BayeScan analyses. We included all environmental variables normalized relative to a reference, and standardized data by dividing each sampling site’s reference value by the population standard deviation. For salinity, we selected two different values as reference: 9 practical salinity units (PSU) as this is known to be the internal osmolality of teleosts, irrespective of environmental salinity [[9](#_ENREF_9)]; and 34 PSU as the environmental salinity of the ancestral state. For average annual SST, we set 8°C as a reference as sand lance larvae are known to be most abundant at water temperatures >7°C. For all other environmental variables, we used the average as reference. We selected the *q*-value as a test statistic to test for significance, and included only those SNPs with a *q*-value < 0.05 in two independent analyses per environmental variable.

We further analyzed the same marker set with BayEnv v.2 [[10](#_ENREF_10)]. This approach controls for demographic effects by using a covariance matrix based on neutral markers when estimating correlations between environmental parameters and genetic differentiation [[11](#_ENREF_11)]. We first estimated a covariance matrix based on neutral SNPs using 100,000 iterations, then tested a set of environmental variables (Table 1) for association with genetic variation, again using 100,000 iterations and three independent analyses. SNPs were identified as outliers in BayEnv2 only if they were very strongly (Bayes Factor 32-100) or decisively (Bayes Factor 100- inf) significant in all three runs according to Jeffreys’ interpretation [[12](#_ENREF_12), [13](#_ENREF_13)].

**Microbial 16S library preparation and amplicon sequencing**

We employed a two-step PCR amplification approach for microbial 16S library preparation and amplified the V3-V4-regions of the bacterial 16S rRNA gene. Total PCR reaction volume for PCR I was 20 μL and consisted of 12μL AccuPrime SuperMix II, 1.5 μL of each forward and reverse primer, 3 μL H_2_O, and 2 μL DNA. PCR conditions were set to an initial denaturation cycle at 95°C for 2 minutes; followed by 38 cycles, each at 95°C for 15s, 55°C for 15s, 68°C for 40s; and a final single extension step at 68°C for 4 minutes.

For PCR II, the total PCR reaction volume was 28 μL, consisting of 12μL AccuPrime SuperMixII, 2 μL of each forward and reverse Nextera^TM^ XT index primers (Illumina Inc., San Diego, US), 7 μL H_2_O, and 5 μL PCR I product. The PCR conditions were as follows: a single initial denaturation cycle at 98°C for 1 minute, followed by 13 cycles each at 98°C for 10s, 55°C for 20s, 68°C for 40s, and a final single extension step at 68°C for 5 minutes. PCR amplification products were cleaned using Agencourt AMPure™ XP magnetic beads following the manufacturer’s protocol (Beckman Coulter Inc., Brea, USA). The PCR products were size-fragmented by gel electrophoresis on a 2% agarose gel, visualized by UV-light exposure, and pooled at equimolar ratios as determined by a Qubit^TM^ 2.0 Fluorometer in a final volume of 20 μL. Negative controls from DNA extraction and amplification steps and were included in the final sequencing library. The PCR amplification products were sequenced as 250bp paired-end sequencing on an Illumina MiSeq™ 1.9 (Illumina Inc., San Diego, US) with 20% phiX spike-in according to the manufacturer’s specifications.

**Microbial 16S data filtration and Operational Taxonomic Unit (OTU) clustering**

Barcode- and primer sequences were stripped from the reads following merging of forward and reverse sequences and removal of any sequence pair <400bp. We then quality-filtered data with a maximum expected error rate of 0.5 and excluded singletons. Possible chimeric sequences were identified using the RDP Gold reference database v.9 [[14](#_ENREF_14)] and were also excluded from the dataset. We then identified operational taxonomic units (OTUs) when the sequence identity level exceeded 97% using the UPARSE algorithm, which constructs representative *de novo* OTU sequences from amplicon data [[15](#_ENREF_15)]. The OTU table was then built by mapping the remaining reads back to OTUs. Finally, taxonomy was assigned to each OTU using the software LCAClassifier v.2.0.4 and the SilvaMod reference database [[16](#_ENREF_16)] with 50 database matches per OTU, and any eukaryotic OTUs were removed. In order to account for differential abundance and read depth in our data, we normalized our dataset with the cumulative-sum scaling (CSS) method [[17](#_ENREF_17)]. As suggested by the authors, we excluded any samples with <1000 reads and in addition used the relative abundances of OTUs per sample for downstream analyses.

**Figure S1: Number of individuals of either species that has < than a certain amount of missing SNP data.**

**Table S2: Pairwise *F_ST_* estimates for *A. tobianus* (A) and *H. lanceolatus* (B). Significant values are displayed in bold (*P* < 0.05). For sampling site abbreviations, see Fig. 1.**

| **(a)** | **TE** | **SB** | **HR** | **LA** | **EB** | **HB** | **HK** | **KB** | **FB** | **BH** | **ÅL** |
| --- | --- | --- | --- | --- | --- | --- | --- | --- | --- | --- | --- |
| **TE** |  |  |  |  |  |  |  |  |  |  |  |
| **SB** | 0.001 |  |  |  |  |  |  |  |  |  |  |
| **HR** | 0 | 0.001 |  |  |  |  |  |  |  |  |  |
| **LA** | **0.005** | **0.005** | **0.005** |  |  |  |  |  |  |  |  |
| **EB** | **0.008** | **0.009** | **0.007** | 0 |  |  |  |  |  |  |  |
| **HB** | **0.006** | **0.006** | **0.004** | 0 | 0 |  |  |  |  |  |  |
| **HK** | **0.011** | **0.009** | **0.007** | 0.001 | 0 | 0.001 |  |  |  |  |  |
| **KB** | **0.016** | **0.018** | **0.013** | **0.008** | **0.006** | **0.008** | **0.005** |  |  |  |  |
| **FB** | **0.028** | **0.03** | **0.024** | **0.017** | **0.015** | **0.018** | **0.012** | 0.001 |  |  |  |
| **BH** | **0.028** | **0.029** | **0.024** | **0.012** | **0.009** | **0.012** | **0.008** | 0.001 | 0.001 |  |  |
| **ÅL** | **0.036** | **0.038** | **0.032** | **0.023** | **0.02** | **0.023** | **0.017** | **0.004** | **0.001** | 0.002 |  |
| **BӦ** | **0.039** | **0.041** | **0.035** | **0.022** | **0.019** | **0.022** | **0.017** | **0.013** | **0.014** | **0.008** | **0.009** |

| **(b)** | **TE** | **SA** | **HR** | **SHA** | **NWH** | **HØ** | **FB** |
| --- | --- | --- | --- | --- | --- | --- | --- |
| **TE** |  |  |  |  |  |  |  |
| **SA** | 0.002 |  |  |  |  |  |  |
| **HR** | 0 | 0.001 |  |  |  |  |  |
| **SHA** | **0.004** | **0.002** | **0.002** |  |  |  |  |
| **NWH** | 0 | 0 | 0 | 0.001 |  |  |  |
| **HØ** | **0.012** | **0.011** | **0.012** | **0.012** | **0.007** |  |  |
| **FB** | **0.039** | **0.037** | **0.038** | **0.039** | **0.032** | **0.007** |  |
| **BH** | **0.039** | **0.038** | **0.038** | **0.039** | **0.032** | **0.008** | 0 |

**Table S3: CV error and Log-likelihood from five independent Admixture** **analyses for *A. tobianus* and *H. lanceolatus*. * denotes the K with the lowest CV error/log-likelihood.**

| ***A. tobianus*** | |  |  |  | ***H. lanceolatus*** | |  |  |
| --- | --- | --- | --- | --- | --- | --- | --- | --- |
|  |  |  |  |  |  |  |  |  |
| **CV error** | **K=2** | **K=3*** | **K=4** |  | **CV error** | **K=2*** | **K=3** | **K=4** |
| **seed1** | 0.52252 | 0.52128 | 0.52471 |  | **seed1** | 0.51018 | 0.51968 | 0.53437 |
| **seed2** | 0.52253 | 0.52121 | 0.52418 |  | **seed2** | 0.51062 | 0.52197 | 0.53204 |
| **seed3** | 0.52251 | 0.52142 | 0.52395 |  | **seed3** | 0.51021 | 0.52268 | 0.53108 |
| **seed4** | 0.52268 | 0.52128 | 0.52456 |  | **seed4** | 0.51023 | 0.52153 | 0.53029 |
| **seed5** | 0.52242 | 0.52125 | 0.52455 |  | **seed5** | 0.50997 | 0.52106 | 0.53037 |
| **Average** | **0.522532** | **0.521288** | **0.52439** |  | **Average** | **0.510242** | **0.521384** | **0.53163** |

**Figure S2: Principal Component analysis (PCA) displaying the dissimilarity between sampling sites in *A. tobianus* (left) and *H. lanceolatus* (right).**


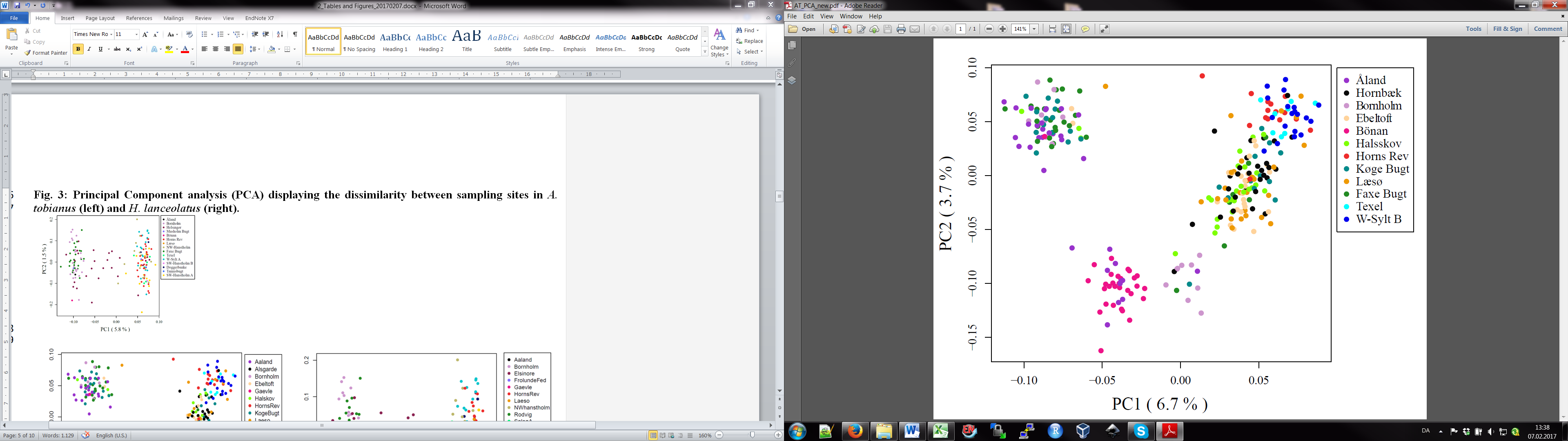

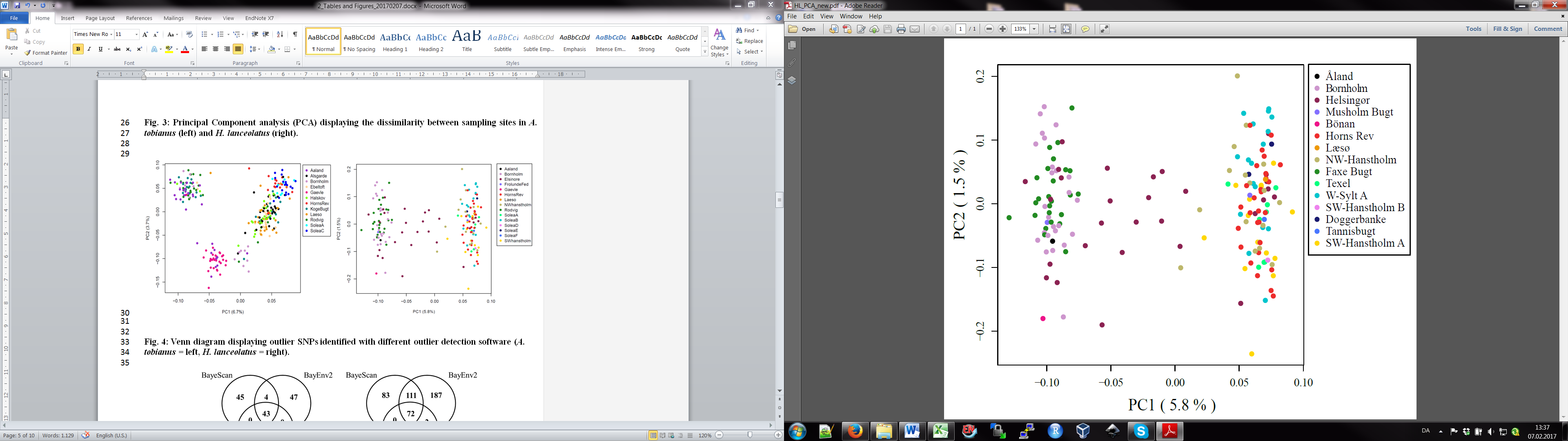


**Table S4a: SNPs which were significant in all three outlier analyses for at least one parameter in *A. tobianus*, and which exhibited a clinal pattern in their allele frequencies along the environmental gradient. BayeScan: FDR = 0.01; BayEnv2: Bayes Factor 32 - 100 = *, Bayes Factor 100 - inf = **; BayeScEnv: q-values < 0.001 = ***; < 0.01 = **; < 0.05 = *.**

|  | **BayeScan** | **BayEnv2** | | |  |  |  |  |  |  |  | **BayeScEnv** | | |  |  |  |  |  |  |  |  |  |
| --- | --- | --- | --- | --- | --- | --- | --- | --- | --- | --- | --- | --- | --- | --- | --- | --- | --- | --- | --- | --- | --- | --- | --- |
| **SNP ID** | **Sign SNPs** | **AB** | **AP** | **BC** | **BP** | **GB** | **VM** | **Sal_av** | **T_av** | **T_max** | **T_min** | **AB** | **AP** | **BC** | **BP** | **GP** | **VM** | **T_R8** | **T_Rav** | **T_CoV** | **T_max_Rav** | **T_min_Rav** | **SalR34** |
| **TP23944** | ** | ** | ** |  |  | ** |  |  |  |  |  |  |  |  |  |  |  |  |  |  | * | * |  |
| **TP30490** | ** |  | * |  |  | ** |  |  |  |  |  | * |  |  |  | * |  |  | ** | * | * | ** |  |
| **TP30521** | ** | ** | * |  |  | * |  |  |  |  |  | * | ** |  | * | ** |  |  |  | *** | *** | ** |  |
| **TP33338** | ** | ** | * |  |  |  |  |  |  |  |  | * | ** |  | * | ** |  |  |  | *** | *** | ** |  |
| **TP36069** | ** | ** | * |  |  | * |  |  |  |  |  |  |  |  | ** |  |  |  |  | ** | ** |  | * |
| **TP37263** | ** | ** | ** |  |  | ** | ** |  |  |  |  |  |  |  |  |  |  |  |  | * | * | * |  |
| **TP56055** | ** | ** | ** | * |  | ** | * |  |  |  |  | * | * |  |  | * |  |  | * | * | * | ** |  |
| **TP57964** | ** | ** | ** | * | * | ** | ** | ** |  |  | ** | * | *** | * | *** | ** | * |  |  | *** | *** | *** |  |
| **TP58792** | ** | ** | ** |  |  | ** |  |  |  |  |  | ** | ** |  |  | ** |  |  | * | * | ** | *** |  |
| **TP59594** | ** | ** | ** |  |  | ** |  |  |  |  |  |  | * |  |  | * |  |  |  | * | ** |  |  |
| **TP65841** | ** | ** | ** | * |  | ** | ** |  |  |  |  |  |  |  |  |  |  |  |  |  | * | * |  |
| **TP67763** | ** | ** | ** |  |  | ** |  | * |  |  |  | * | ** |  | *** | ** | * |  |  | ** | *** | ** |  |
| **TP67909** | ** | ** | ** |  |  | * |  | * |  |  | ** |  | * | * | * | * | * |  |  |  | ** |  |  |
| **TP70552** | ** | ** | ** |  |  | ** | * |  |  |  |  | ** | ** |  |  | ** |  |  | * | * | * | ** |  |
| **TP75256** | ** | ** | ** | ** |  | ** | ** | ** |  |  | ** | *** | *** |  | ** | *** |  |  |  |  | ** | *** |  |
| **TP76138** | ** | ** | ** |  |  | ** |  |  |  |  |  |  |  |  |  |  |  |  |  |  | * | * |  |
| **TP81121** | ** | ** | ** | ** |  | ** | ** | ** |  |  |  | ** | *** |  | * | *** |  |  |  |  | * |  |  |
| **TP87364** | ** | ** | ** | ** | * | ** | ** | ** | * | ** | ** | ** | *** |  | * | *** |  |  |  |  | * |  |  |
| **TP91275** | ** | ** | ** |  |  | ** |  | ** |  |  |  |  | * |  | * | * |  |  |  | ** | ** | * | * |
| **TP91333** | ** | ** | ** | * |  | ** | * | ** |  |  | ** | * | *** | * | *** | ** | * |  |  | *** | *** | *** |  |
| **TP96745** | ** | ** |  |  |  |  |  |  |  |  |  |  |  |  |  |  |  |  |  |  |  | * |  |
| **TP99151** | ** | ** | ** |  | ** |  |  |  | ** |  |  |  | ** | ** | ** | * | ** |  |  | *** | *** |  | ** |

AB = Actinobacteria, AP = Alphaproteobacteria, BC = Bacteroidetes, BP = Betaproteobacteria, GP = Gammaproteobacteria, VM = Verrucomicrobia, Sal_av = average annual salinity, T_av = average annual SST, T_max = maximal annual SST, T_min = minimum annual SST, T_R8 = average annual SST standardized and referenced to 8°C, T_Rav = average annual SST standardized and average used as reference, T_CoV = average annual SST standardized and the coefficient of variance used as reference, T_max_Rav = maximum annual SST standardized and average used as reference, T_min_Rav= minimum annual SST standardized and average used as reference, SalR34 = average annual salinity standardized and 34PSU used as reference

**Table S4b: SNPs which were significant in all three outlier analyses for at least one parameter in *H. lanceolatus*, and which exhibited a clinal pattern in their allele frequencies along the environmental gradient. BayeScan: FDR = 0.01; BayEnv2: Bayes Factor 32 - 100 = *, Bayes Factor 100 - inf = **; BayeScEnv: q-values < 0.001 = ***; < 0.01 = **; < 0.05 = *.**

|  | **BayeScan** | **BayEnv2** | **BayeScEnv** | |  |  |  |  |
| --- | --- | --- | --- | --- | --- | --- | --- | --- |
| **SNP ID** | **sign SNPs** | **Sal_av** | **SalR9** | **T_R8** | **T_Rav** | **T_CoV** | **T_max_Rav** | **T_min_Rav** |
| **TP12524** | ** | ** |  |  |  | * |  |  |
| **TP12818** | ** | ** |  |  | * | * |  |  |
| **TP158** | ** | ** | ** | ** | *** | *** | *** | ** |
| **TP1597** | ** | ** | * | * | ** | ** |  |  |
| **TP1696** | ** | ** |  |  |  | * | ** |  |
| **TP18227** | ** | ** | * | * | ** | ** | * | * |
| **TP18576** | ** | ** | * | * | ** | ** | ** | ** |
| **TP20677** | ** | ** | *** | * | *** | *** | *** | ** |
| **TP21309** | ** | ** | ** | * | *** | *** | *** | *** |
| **TP2253** | ** | ** | * |  | ** | * | * | * |
| **TP24905** | ** | ** | *** | * | *** | ** | ** | *** |
| **TP27390** | ** | ** | ** | * | *** | *** | *** | ** |
| **TP29888** | ** | ** |  | * | * | * |  |  |
| **TP38895** | ** | ** | * |  | ** | ** | ** | * |
| **TP41597** | ** | ** | ** | * | *** | *** | *** | ** |
| **TP42069** | ** | ** |  |  | * | * | * |  |
| **TP45414** | ** | ** |  |  | * | * | ** |  |
| **TP51182** | ** | ** | * |  | ** | ** | ** | * |
| **TP5289** | ** | ** |  |  | * |  |  |  |
| **TP53061** | ** | ** | * | * | ** | ** | * |  |
| **TP57120** | ** | ** | * |  | * | * | * | * |
| **TP58658** | ** | ** |  | * | * |  |  |  |
| **TP60353** | ** | ** |  |  | * | ** | ** |  |
| **TP62255** | ** | ** | ** | * | *** | *** | *** | ** |
| **TP64203** | ** | ** |  |  | * |  |  | * |
| **TP655** | ** | ** | ** | ** | *** | *** | *** | ** |
| **TP6574** | ** | ** | * |  | * | * | * | * |
| **TP7133** | ** | ** | *** | * | *** | ** | ** | *** |
| **TP9434** | ** | ** | ** | * | ** | ** | ** | ** |

Sal_av = average annual salinity, SalR9 = average annual salinity standardized and 9PSU used as reference, T_R8 = average annual SST standardized and referenced to 8°C, T_Rav = average annual SST standardized and average used as reference, T_CoV = average annual SST standardized and the coefficient of variance used as reference, T_max_Rav = maximum annual SST standardized and average used as reference, T_min_Rav= minimum annual SST standardized and average used as reference

**Figure S3a: Allele frequency plots of each outlier locus for *A. tobianus*. The allele frequency of the respective major allele is displayed. Outlier locus names are displayed in red. Sampling sites are ordered from North Sea (left) to Baltic Sea (right).**


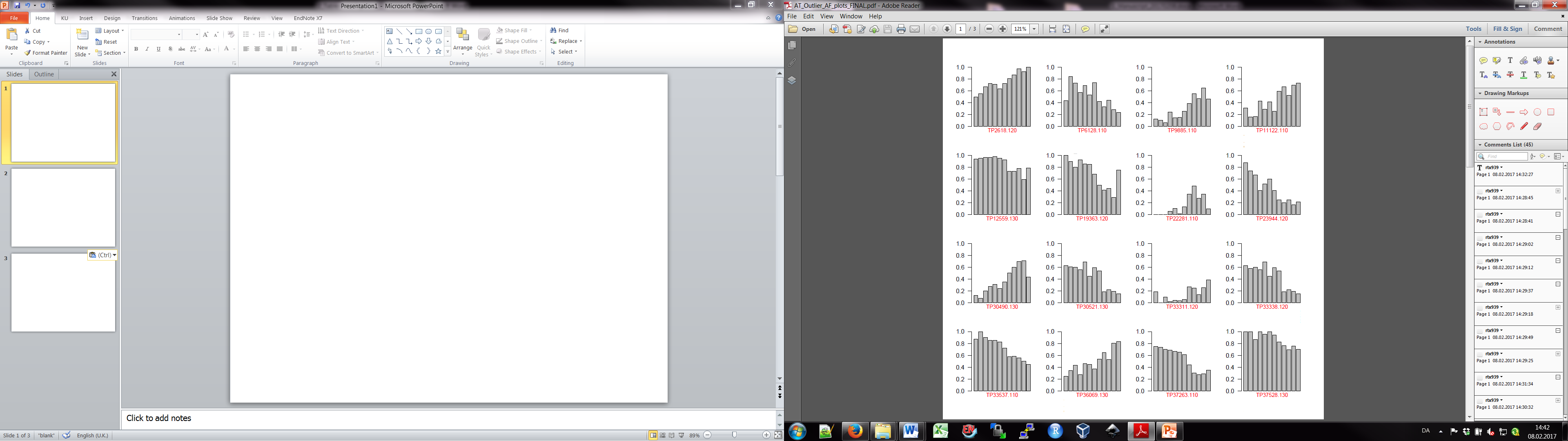


**Texel**

**W-Sylt B**

**Horns Rev**

**Læsø**

**Ebeltoft**

**Hornbæk**

**Halsskov**

**Køge Bugt**

**Faxe Bugt**

**Bornholm**

**Åland**

**Bönan**

**Texel**

**W-Sylt B**

**Horns Rev**

**Læsø**

**Ebeltoft**

**Hornbæk**

**Halsskov**

**Køge Bugt**

**Faxe Bugt**

**Bornholm**

**Åland**

**Bönan**

**Texel**

**W-Sylt B**

**Horns Rev**

**Læsø**

**Ebeltoft**

**Hornbæk**

**Halsskov**

**Køge Bugt**

**Faxe Bugt**

**Bornholm**

**Åland**

**Bönan**

**Texel**

**W-Sylt B**

**Horns Rev**

**Læsø**

**Ebeltoft**

**Hornbæk**

**Halsskov**

**Køge Bugt**

**Faxe Bugt**

**Bornholm**

**Åland**

**Bönan**


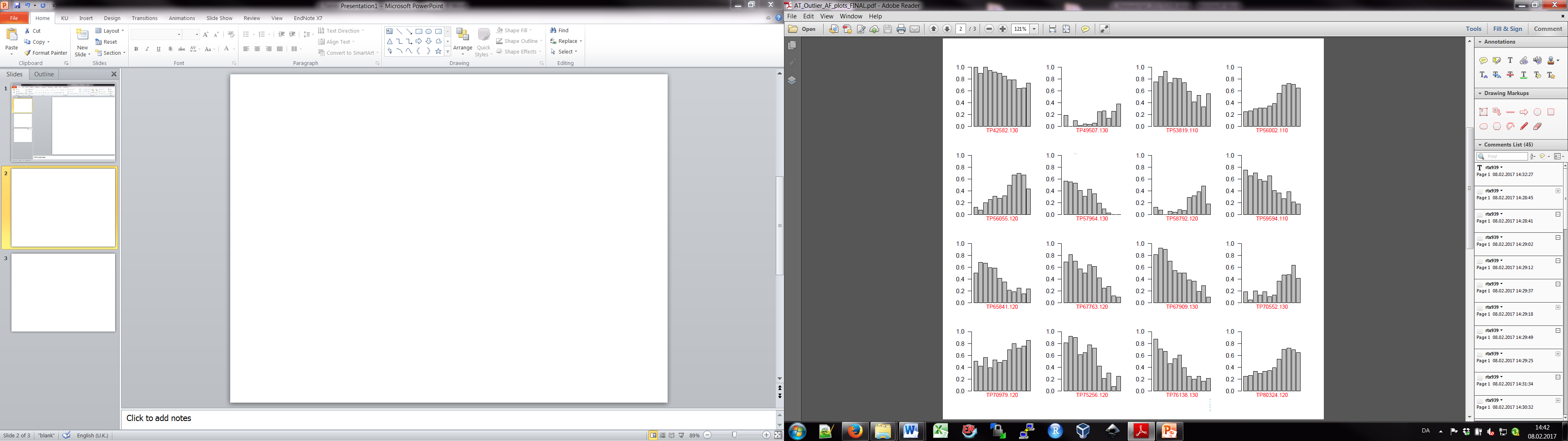


**Texel**

**W-Sylt B**

**Horns Rev**

**Læsø**

**Ebeltoft**

**Hornbæk**

**Halsskov**

**Køge Bugt**

**Faxe Bugt**

**Bornholm**

**Åland**

**Bönan**

**Texel**

**W-Sylt B**

**Horns Rev**

**Læsø**

**Ebeltoft**

**Hornbæk**

**Halsskov**

**Køge Bugt**

**Faxe Bugt**

**Bornholm**

**Åland**

**Bönan**

**Texel**

**W-Sylt B**

**Horns Rev**

**Læsø**

**Ebeltoft**

**Hornbæk**

**Halsskov**

**Køge Bugt**

**Faxe Bugt**

**Bornholm**

**Åland**

**Bönan**

**Texel**

**W-Sylt B**

**Horns Rev**

**Læsø**

**Ebeltoft**

**Hornbæk**

**Halsskov**

**Køge Bugt**

**Faxe Bugt**

**Bornholm**

**Åland**

**Bönan**


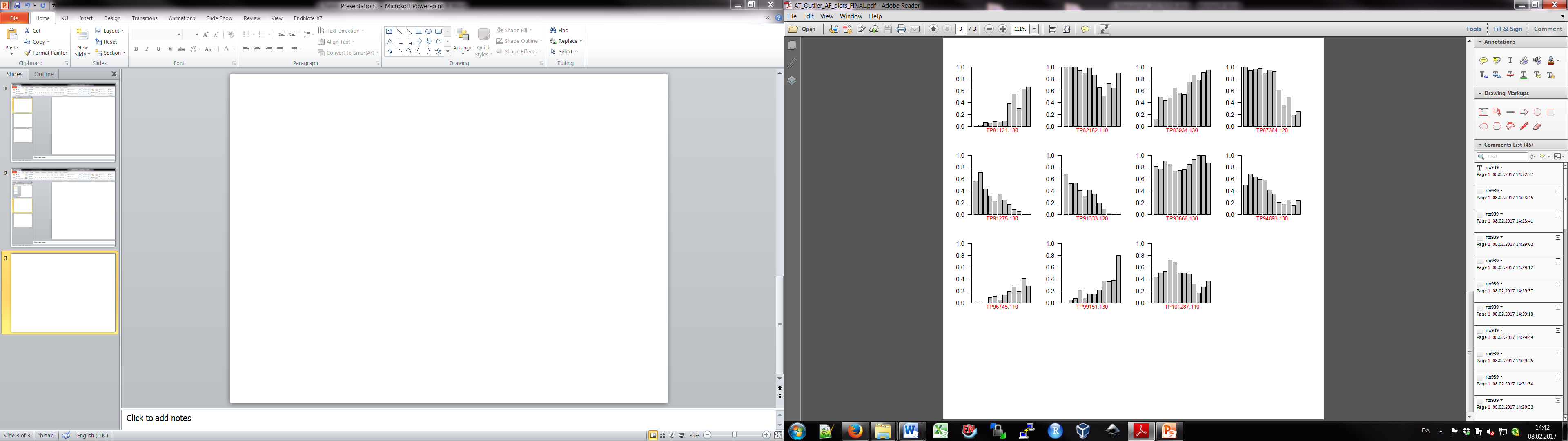


**Texel**

**W-Sylt B**

**Horns Rev**

**Læsø**

**Ebeltoft**

**Hornbæk**

**Halsskov**

**Køge Bugt**

**Faxe Bugt**

**Bornholm**

**Åland**

**Bönan**

**Texel**

**W-Sylt B**

**Horns Rev**

**Læsø**

**Ebeltoft**

**Hornbæk**

**Halsskov**

**Køge Bugt**

**Faxe Bugt**

**Bornholm**

**Åland**

**Bönan**

**Texel**

**W-Sylt B**

**Horns Rev**

**Læsø**

**Ebeltoft**

**Hornbæk**

**Halsskov**

**Køge Bugt**

**Faxe Bugt**

**Bornholm**

**Åland**

**Bönan**

**Texel**

**W-Sylt B**

**Horns Rev**

**Læsø**

**Ebeltoft**

**Hornbæk**

**Halsskov**

**Køge Bugt**

**Faxe Bugt**

**Bornholm**

**Åland**

**Bönan**

**Figure S3b: Allele frequency plots of each outlier locus for *H. lanceolatus*. The allele frequency of the respective major allele is displayed. Outlier locus names are displayed in red. Sampling sites are ordered from North Sea (left) to Baltic Sea (right).**


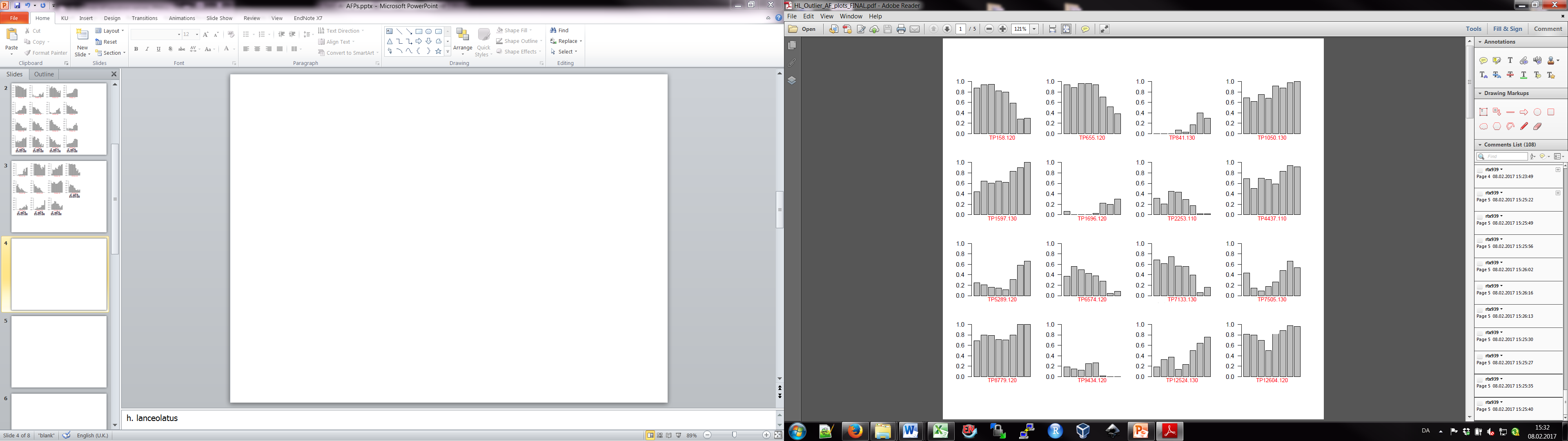


**Texel**

**W-Sylt A**

**Horns Rev**

**SW-Hanstholm A**

**NW-Hanstholm**

**Helsingør**

**Faxe Bugt**

**Bornholm**

**Texel**

**W-Sylt A**

**Horns Rev**

**SW-Hanstholm A**

**NW-Hanstholm**

**Helsingør**

**Faxe Bugt**

**Bornholm**

**Texel**

**W-Sylt A**

**Horns Rev**

**SW-Hanstholm A**

**NW-Hanstholm**

**Helsingør**

**Faxe Bugt**

**Bornholm**

**Texel**

**W-Sylt A**

**Horns Rev**

**SW-Hanstholm A**

**NW-Hanstholm**

**Helsingør**

**Faxe Bugt**

**Bornholm**


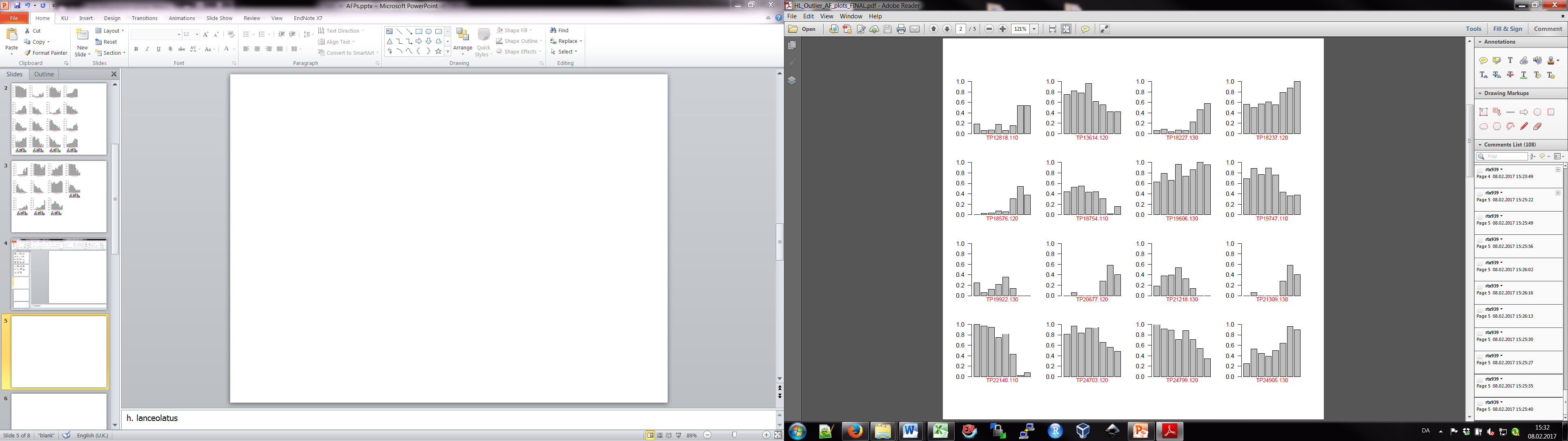


**Texel**

**W-Sylt A**

**Horns Rev**

**SW-Hanstholm A**

**NW-Hanstholm**

**Helsingør**

**Faxe Bugt**

**Bornholm**

**Texel**

**W-Sylt A**

**Horns Rev**

**SW-Hanstholm A**

**NW-Hanstholm**

**Helsingør**

**Faxe Bugt**

**Bornholm**

**Texel**

**W-Sylt A**

**Horns Rev**

**SW-Hanstholm A**

**NW-Hanstholm**

**Helsingør**

**Faxe Bugt**

**Bornholm**

**Texel**

**W-Sylt A**

**Horns Rev**

**SW-Hanstholm A**

**NW-Hanstholm**

**Helsingør**

**Faxe Bugt**

**Bornholm**


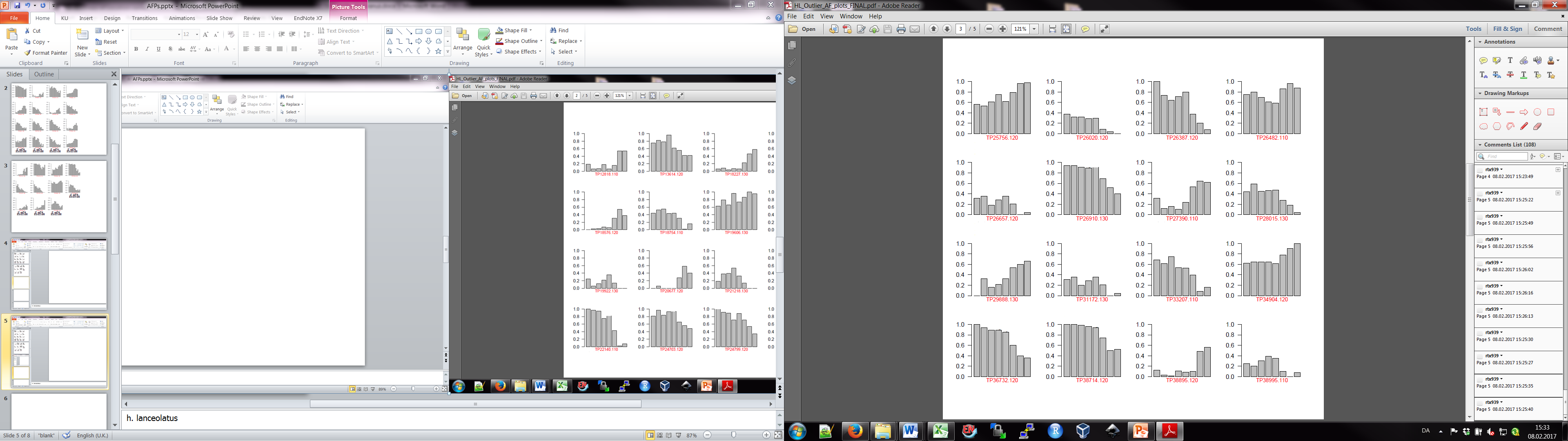


**Texel**

**W-Sylt A**

**Horns Rev**

**SW-Hanstholm A**

**NW-Hanstholm**

**Helsingør**

**Faxe Bugt**

**Bornholm**

**Texel**

**W-Sylt A**

**Horns Rev**

**SW-Hanstholm A**

**NW-Hanstholm**

**Helsingør**

**Faxe Bugt**

**Bornholm**

**Texel**

**W-Sylt A**

**Horns Rev**

**SW-Hanstholm A**

**NW-Hanstholm**

**Helsingør**

**Faxe Bugt**

**Bornholm**

**Texel**

**W-Sylt A**

**Horns Rev**

**SW-Hanstholm A**

**NW-Hanstholm**

**Helsingør**

**Faxe Bugt**

**Bornholm**


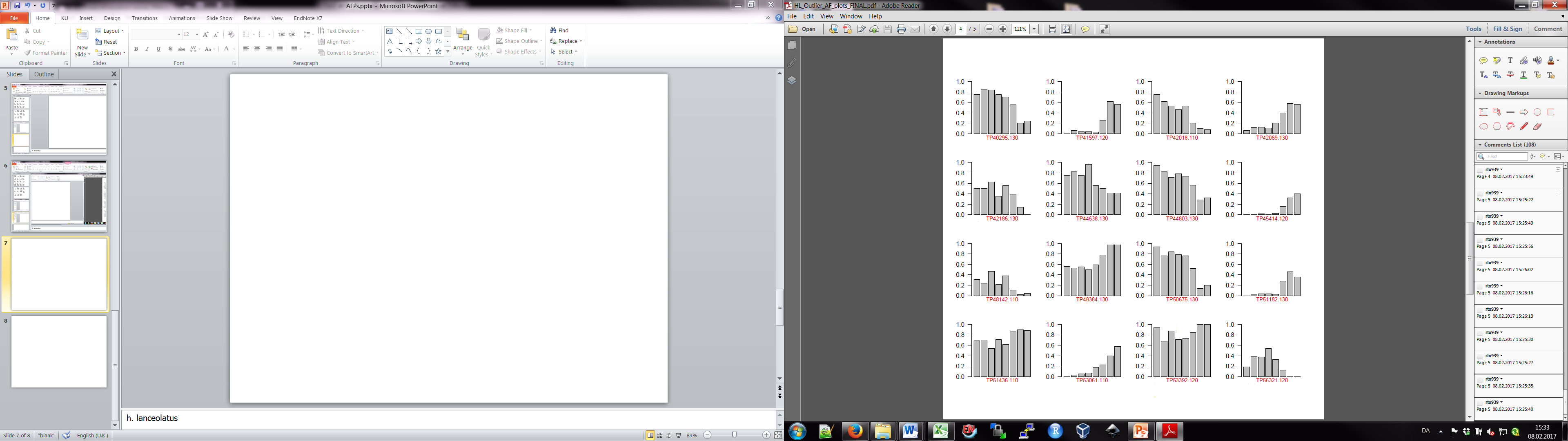


**Texel**

**W-Sylt A**

**Horns Rev**

**SW-Hanstholm A**

**NW-Hanstholm**

**Helsingør**

**Faxe Bugt**

**Bornholm**

**Texel**

**W-Sylt A**

**Horns Rev**

**SW-Hanstholm A**

**NW-Hanstholm**

**Helsingør**

**Faxe Bugt**

**Bornholm**

**Texel**

**W-Sylt A**

**Horns Rev**

**SW-Hanstholm A**

**NW-Hanstholm**

**Helsingør**

**Faxe Bugt**

**Bornholm**

**Texel**

**W-Sylt A**

**Horns Rev**

**SW-Hanstholm A**

**NW-Hanstholm**

**Helsingør**

**Faxe Bugt**

**Bornholm**


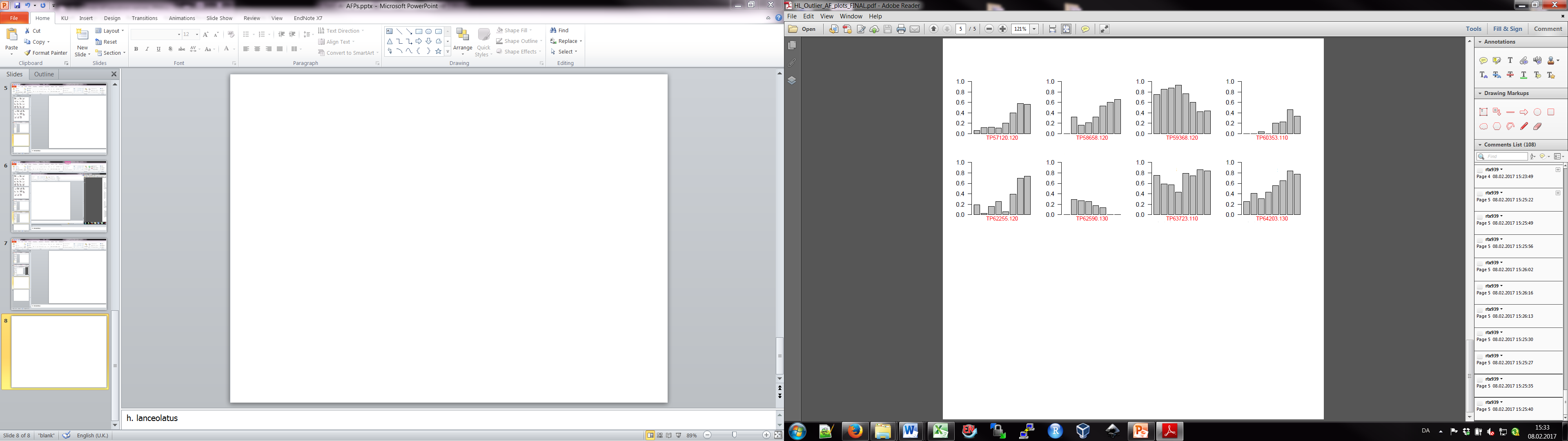


**Texel**

**W-Sylt A**

**Horns Rev**

**SW-Hanstholm A**

**NW-Hanstholm**

**Helsingør**

**Faxe Bugt**

**Bornholm**

**Texel**

**W-Sylt A**

**Horns Rev**

**SW-Hanstholm A**

**NW-Hanstholm**

**Helsingør**

**Faxe Bugt**

**Bornholm**

**Texel**

**W-Sylt A**

**Horns Rev**

**SW-Hanstholm A**

**NW-Hanstholm**

**Helsingør**

**Faxe Bugt**

**Bornholm**

**Texel**

**W-Sylt A**

**Horns Rev**

**SW-Hanstholm A**

**NW-Hanstholm**

**Helsingør**

**Faxe Bugt**

**Bornholm**

**Table S5: Read depth per sample pre- and post-normalization treatment for 16S datasets.**

| **Species** | **Sampling Site** | **Sample ID** | **Read Depth before Normalization** | **CSS-normalized Read Depth** |
| --- | --- | --- | --- | --- |
| ***A. tobianus*** | Faxe Bugt | P611034 | 1051 | 72.118 |
|  |  | P611035 | 2667 | 157.0389 |
|  |  | P611037 | 4464 | 274.8606 |
|  |  | P611039 | 2381 | 213.5958 |
|  |  | P611040 | 2098 | 206.6746 |
|  |  | P611041 | 1915 | 145.4706 |
|  |  | P611042 | 2086 | 117.1675 |
|  | Halsskov | P611074 | 5578 | 118.7134 |
|  |  | P611075 | 5072 | 170.8409 |
|  |  | P611076 | 5299 | 97.8954 |
|  |  | P611077 | 2463 | 131.6985 |
|  |  | P611078 | 7377 | 182.7471 |
|  |  | P611079 | 8605 | 195.9542 |
|  |  | P611080 | 6334 | 236.0088 |
|  |  | P611195 | 7808 | 123.9369 |
|  |  | P611196 | 4285 | 118.9863 |
|  |  | P611197 | 5262 | 171.2092 |
|  | Bönan | P611263 | 14098 | 358.2138 |
|  |  | P611264 | 14916 | 461.0863 |
|  |  | P611265 | 16892 | 255.1114 |
|  |  | P611266 | 25352 | 313.3477 |
|  |  | P611267 | 19161 | 302.2921 |
|  |  | P611268 | 17958 | 391.7743 |
|  |  | P611269 | 13137 | 379.9862 |
|  |  | P611270 | 17703 | 244.9897 |
|  |  | P611272 | 8428 | 76.2335 |
|  | Ebeltoft | P611287 | 1124 | 139.774 |
|  |  | P611288 | 2349 | 181.9596 |
|  |  | P611289 | 4434 | 98.1227 |
|  |  | P611290 | 15022 | 102.556 |
|  |  | P611291 | 5083 | 93.9639 |
| ***H. lanceolatus*** | SW-Hanstholm A | P611093b | 3924 | 114.7141 |
|  |  | P611110 | 10439 | 73.5825 |
|  |  | P611112 | 8712 | 90.11 |
|  |  | P611113 | 7481 | 160.9203 |
|  |  | P611114 | 2005 | 259.3116 |
|  |  | P611115 | 3667 | 108.4447 |
|  |  | P611116 | 4061 | 214.7052 |
|  |  | P611117 | 3241 | 178.6229 |
|  |  | P611118 | 6099 | 133.371 |
|  |  | P611119 | 9070 | 75.6291 |
|  | Helsingør | P611229 | 6767 | 66.5764 |
|  |  | P611231 | 2288 | 67.2825 |
|  |  | P611233 | 12492 | 229.389 |
|  |  | P611234 | 2979 | 134.4168 |
|  |  | P611235 | 1891 | 55.9113 |
|  |  | P611236 | 3169 | 148.2678 |
|  |  | P611237 | 4687 | 55.8623 |
|  |  | P611238 | 4111 | 53.1344 |
|  |  | P611239 | 1348 | 70.4604 |

**Table S6: Permanova R^2^ results and significance levels to identify which parameters have a significant influence on the variation in gut microbiome composition. Data for the parameters Alphaproteobacteria (Alphapro), Verrucomicrobia (Verrucom), Bacteroidetes (Bacteroi), Betaproteobacteria (Betaprot), Actinobacteria (Actinoba), and Gammaproteobacteria (Gammapro) are taken from** [**Hu Y, Karlson B, Charvet S and Andersson AF [1]**](#_ENREF_1)**. *P*-values < 0.001 = ***; < 0.01 = **; < 0.05 = ***

| ***A. tobianus*** | |  |  |  |  |  |  |  |
| --- | --- | --- | --- | --- | --- | --- | --- | --- |
|  | **Parameter** | **Df** | **SumsOfSqs** | **MeanSqs** | **F.Model** | **R^2^** | **Pr(>F)** | **Sign** |
|  | **Date** | 1 | 0.8555 | 0.85549 | 3.101 | 0.09971 | 0.004 | ** |
|  | **Distance** | 1 | 2.1224 | 2.12241 | 9.2026 | 0.24736 | 0.001 | *** |
|  | **Std_length** | 1 | 0.6986 | 0.69858 | 2.4818 | 0.08142 | 0.019 | * |
|  | **stomach_contents** | 1 | 1.1469 | 1.1469 | 4.3202 | 0.13367 | 0.001 | *** |
|  | **Salinity_annual_ave** | 1 | 2.2061 | 2.20608 | 9.6909 | 0.25712 | 0.001 | *** |
|  | **SST_annual_ave** | 1 | 2.278 | 2.27798 | 10.121 | 0.2655 | 0.001 | *** |
|  | **SST_min** | 1 | 2.0172 | 2.01723 | 8.6063 | 0.23511 | 0.001 | *** |
|  | **SST_max** | 1 | 0.7303 | 0.73028 | 2.6049 | 0.08511 | 0.012 | * |
|  | **SST_Coeff_var** | 1 | 0.739 | 0.73895 | 2.6387 | 0.08612 | 0.011 | * |
|  | **Q_North_AT** | 1 | 1.1244 | 1.12436 | 4.2225 | 0.13104 | 0.001 | *** |
|  | **Alphapro** | 1 | 1.2999 | 1.29989 | 5.3196 | 0.22812 | 0.001 | *** |
|  | **Verrucom** | 1 | 1.0277 | 1.02773 | 3.9608 | 0.18036 | 0.003 | ** |
|  | **Bacteroi** | 1 | 0.9241 | 0.92409 | 3.484 | 0.16217 | 0.002 | ** |
|  | **Betaprot** | 1 | 1.2982 | 1.2982 | 5.3107 | 0.22782 | 0.001 | *** |
|  | **Actinoba** | 1 | 1.4518 | 1.45182 | 6.154 | 0.25478 | 0.001 | *** |
|  | **Gammapro** | 1 | 1.2033 | 1.20328 | 4.8184 | 0.21116 | 0.001 | *** |
|  |  |  |  |  |  |  |  |  |
| ***H. lanceolatus*** | |  |  |  |  |  |  |  |
|  | **Parameter** | **Df** | **SumsOfSqs** | **MeanSqs** | **F.Model** | **R^2^** | **Pr(>F)** | **Sign** |
|  | **Date** | 1 | 0.8973 | 0.89725 | 3.4498 | 0.1977 | 0.001 | *** |
|  | **Distance** | 1 | 0.8973 | 0.89725 | 3.4498 | 0.1977 | 0.001 | *** |
|  | **Std_length** | 1 | 0.9148 | 0.91476 | 3.5341 | 0.20156 | 0.002 | ** |
|  | **stomach_contents** | 2 | 1.0653 | 0.53263 | 1.9936 | 0.23472 | 0.01 |  |
|  | **Salinity_annual_ave** | 1 | 0.8973 | 0.89725 | 3.4498 | 0.1977 | 0.006 | ** |
|  | **SST_annual_ave** | 1 | 0.8973 | 0.89725 | 3.4498 | 0.1977 | 0.001 | *** |
|  | **SST_min** | 1 | 0.8973 | 0.89725 | 3.4498 | 0.1977 | 0.002 | ** |
|  | **SST_max** | 1 | 0.8973 | 0.89725 | 3.4498 | 0.1977 | 0.005 | ** |
|  | **SST_Coeff_var** | 1 | 0.8973 | 0.89725 | 3.4498 | 0.1977 | 0.002 | ** |
|  | **Q_North_HL** | 1 | 0.4794 | 0.47936 | 1.6533 | 0.10562 | 0.093 | . |

**Table S7: Chi^2^ results to test significant changes in proportion of major bacterial taxa in the sand lance guts and in the Baltic water; p-values < 0.001 = ***; < 0.01 = **; < 0.05 = *.** **Arrows indicate an increase or decrease in relative abundance with decreasing salinity.**

| **Taxon** | **Gut** | **Water** |
| --- | --- | --- |
| **Alphapro** | 27.468*** | 18.098*** |
| **Verrucom** | 3.081 | 14.721*** |
| **Bacteroi** | 0.907 | 4.197 |
| **Betaprot** | 2.859 | 6.348* |
| **Actinoba** | 24.414*** | 10.992** |
| **Gammapro** | 119.590*** | 16.146*** |

**Figure S4: Two-dimensional NMDS dissimilarity ordination displaying how the bacterial communities in *A. tobianus* (left) and *H. lanceolatus* (right) relate to one another. Environmental parameters that significantly describe variation in community data based on a Permanova test, and that were not collinear among each other, are displayed as vectors.**


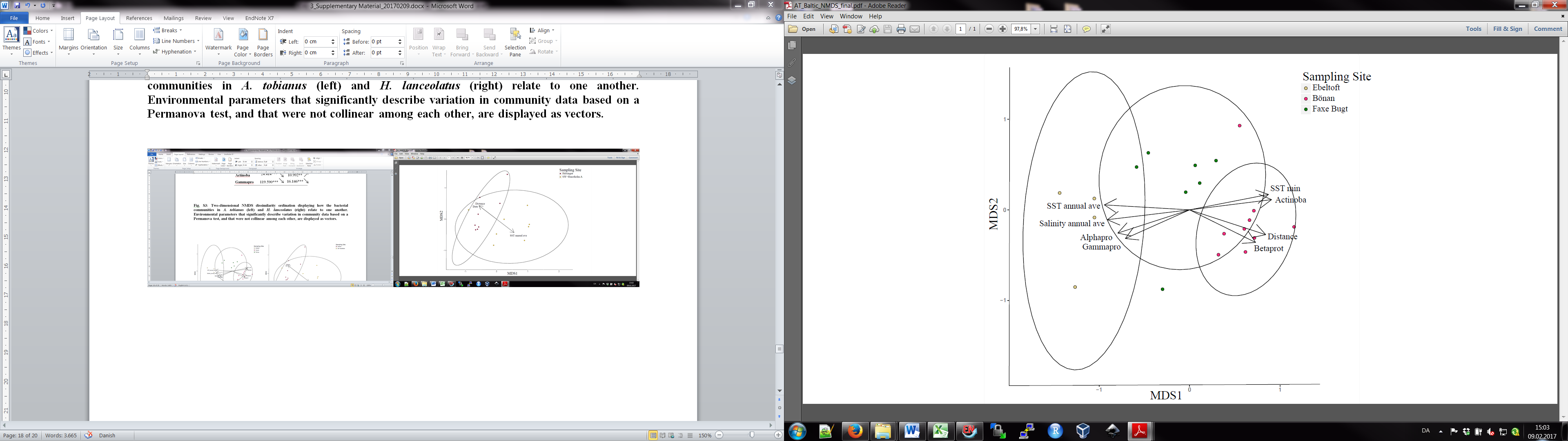

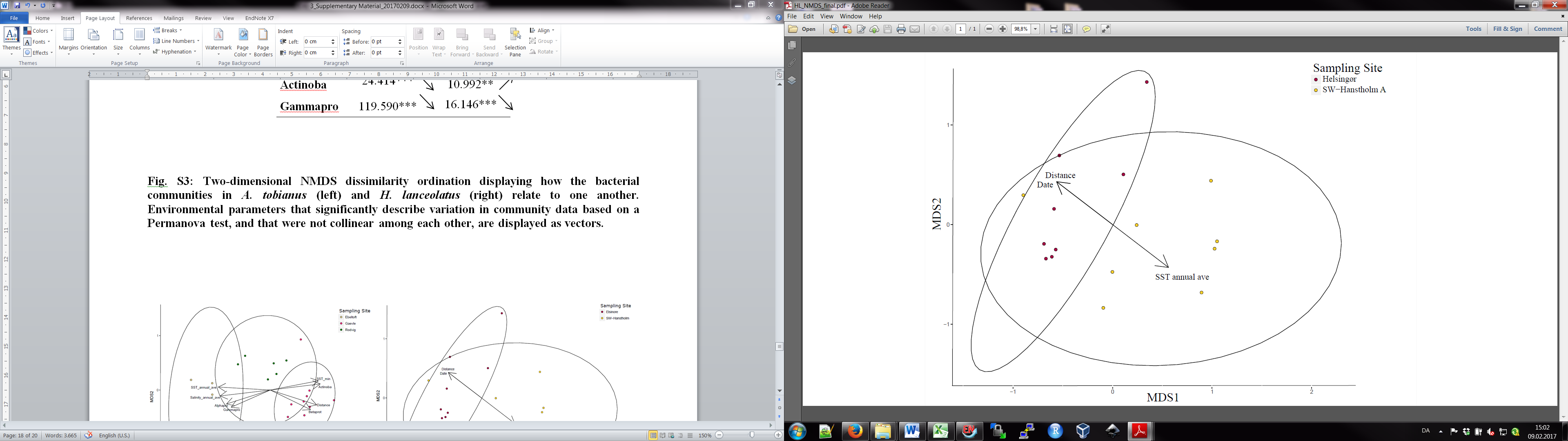


**Figure S5: Constrained analysis of principal coordinates displaying the amount of variation in gut microbial communities that is explained by host species identity. AM = *Ammodytes marinus*, AN = Unknown, AT = *Ammodytes tobianus*, FL = Flounder (Platichthys *flesus*), HL = *Hyperoplus lanceolatus*, PM = *Pomatoschistus minutus*, GA = Gasterosteus aculeatus. Distance refers to the geographical distance from the most Western sampling site. Ellipses encircle fish species with >2 available samples.**


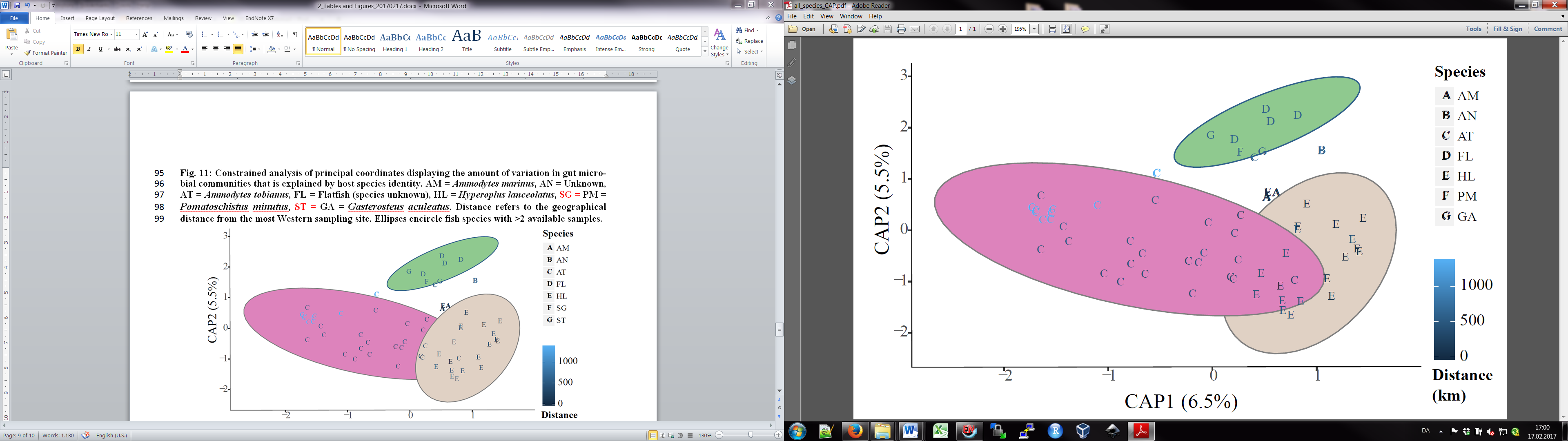


**Figure S6: Bar chart representing the gut microbiome composition by taxonomic order in *A. tobianus* (left and center group representing the two sampling sites Halsskov (left) and Faxe Bugt (right)) and an outgroup consisting of a variety of Baltic fish species (Køge Bugt (right)).**

**References**

1. Hu YOO, Karlson B, Charvet S, Andersson AF: **Diversity of Pico- to Mesoplankton along the 2000 km Salinity Gradient of the Baltic Sea.** *Frontiers in Microbiology* 2016, **7**.

2. Lu F, Lipka AE, Glaubitz J, Elshire R, Cherney JH, Casler MD, Buckler ES, Costich DE: **Switchgrass Genomic Diversity, Ploidy, and Evolution: Novel Insights from a Network-Based SNP Discovery Protocol.** *Plos Genetics* 2013, **9**.

3. Purcell S, Neale B, Todd-Brown K, Thomas L, Ferreira MAR, Bender D, Maller J, Sklar P, de Bakker PIW, Daly MJ, Sham PC: **PLINK: A tool set for whole-genome association and population-based linkage analyses.** *American Journal of Human Genetics* 2007, **81:**559-575.

4. Foll M, Gaggiotti O: **A Genome-Scan Method to Identify Selected Loci Appropriate for Both Dominant and Codominant Markers: A Bayesian Perspective.** *Genetics* 2008, **180:**977-993.

5. Geweke J: **Evaluating the accuracy of sampling-based approaches to calculating posterior moments.** In *Bayesian Statistics 4.* Edited by Bernado M, Berger JO, Dawid AP, Smith AFM. Oxford, UK: Clarendon Press; 1991

6. Gelman A, Rubin DB: **Inference from Iterative Simulation Using Multiple Sequences** *Statistical Science* 1992, **7:**457-511.

7. Plummer M, Best N, Cowles K, Vines K, Sarkar D, Bates D, Almond R, Magusson A: **Output Analysis and Diagnostics for MCMC Version 0.19-1.** 2016.

8. de Villemereuil P, Gaggiotti OE: **A new FST-based method to uncover local adaptation using environmental variables.** *Methods in Ecology and Evolution* 2015, **6:**1248-1258.

9. Kultz D: **Physiological mechanisms used by fish to cope with salinity stress.** *Journal of Experimental Biology* 2015, **218:**1907-1914.

10. Gunther T, Coop G: **Robust Identification of Local Adaptation from Allele Frequencies.** *Genetics* 2013, **195:**205-+.

11. Coop G, Witonsky D, Di Rienzo A, Pritchard JK: **Using Environmental Correlations to Identify Loci Underlying Local Adaptation.** *Genetics* 2010, **185:**1411-1423.

12. Jeffreys H: *Theory of Probability;* 2edn. Oxford: The Clarendon Press; 1948.

13. Robert CP, Chopin N, Rousseau J, Bernardo JM, Gelman A, Kass R, Lindley D, Senn S, Zellner A: **Harold Jeffreys's Theory of Probability Revisited.** *Statistical Science* 2009, **24:**141-194.

14. Cole JR, Wang Q, Fish JA, Chai BL, McGarrell DM, Sun YN, Brown CT, Porras-Alfaro A, Kuske CR, Tiedje JM: **Ribosomal Database Project: data and tools for high throughput rRNA analysis.** *Nucleic Acids Research* 2014, **42:**D633-D642.

15. Edgar RC: **UPARSE: highly accurate OTU sequences from microbial amplicon reads.** *Nature Methods* 2013, **10:**996-+.

16. Lanzen A, Jorgensen SL, Huson DH, Gorfer M, Grindhaug SH, Jonassen I, Ovreas L, Urich T: **CREST - Classification Resources for Environmental Sequence Tags.** *Plos One* 2012, **7**.

17. Paulson JN, Stine OC, Bravo HC, Pop M: **Differential abundance analysis for microbial marker-gene surveys.** *Nature Methods* 2013, **10:**1200-+.
